# Scoring Tools for the Analysis of Infant Respiratory Inductive Plethysmography Signals

**DOI:** 10.1371/journal.pone.0134182

**Published:** 2015-07-28

**Authors:** Carlos Alejandro Robles-Rubio, Gianluca Bertolizio, Karen A. Brown, Robert E. Kearney

**Affiliations:** 1 Department of Biomedical Engineering, McGill University, Montreal, Quebec, Canada; 2 Department of Anesthesia, McGill University Health Centre, Montreal, Quebec, Canada; University of Adelaide, AUSTRALIA

## Abstract

Infants recovering from anesthesia are at risk of life threatening Postoperative Apnea (POA). POA events are rare, and so the study of POA requires the analysis of long cardiorespiratory records. Manual scoring is the preferred method of analysis for these data, but it is limited by low intra- and inter-scorer repeatability. Furthermore, recommended scoring rules do not provide a comprehensive description of the respiratory patterns. This work describes a set of manual scoring tools that address these limitations. These tools include: (i) a set of definitions and scoring rules for 6 mutually exclusive, unique patterns that fully characterize infant respiratory inductive plethysmography (RIP) signals; (ii) RIPScore, a graphical, manual scoring software to apply these rules to infant data; (iii) a library of data segments representing each of the 6 patterns; (iv) a fully automated, interactive formal training protocol to standardize the analysis and establish intra- and inter-scorer repeatability; and (v) a quality control method to monitor scorer ongoing performance over time. To evaluate these tools, three scorers from varied backgrounds were recruited and trained to reach a performance level similar to that of an expert. These scorers used RIPScore to analyze data from infants at risk of POA in two separate, independent instances. Scorers performed with high accuracy and consistency, analyzed data efficiently, had very good intra- and inter-scorer repeatability, and exhibited only minor confusion between patterns. These results indicate that our tools represent an excellent method for the analysis of respiratory patterns in long data records. Although the tools were developed for the study of POA, their use extends to any study of respiratory patterns using RIP (e.g., sleep apnea, extubation readiness). Moreover, by establishing and monitoring scorer repeatability, our tools enable the analysis of large data sets by multiple scorers, which is essential for longitudinal and multicenter studies.

## Introduction

Anesthesia enhances the susceptibility to apnea in infants [1–5], leading to Postoperative Apnea (POA) events that may be life threatening, so infants require continuous cardiorespiratory monitoring [1, 2, 6]. POA events are rare with most occurring in the initial postoperative hours, but a delayed onset, as late as 12 hours after surgery, has been reported [2–4]. Thus, any comprehensive study of POA requires the analysis of long data records.

Measuring infant respiration for extended periods of time requires a sensor that is well tolerated during both sleep and wakefulness. The initial studies of POA monitored respiration with thoracic impedance [2, 7, 8], the sensor of respiration most commonly used clinically in Postanesthesia Care Units (PACU). However, this sensor has important limitations leading to missed apneas, as both obstructive apnea and cardiogenic oscillations may often be misinterpreted as breathing [9]. Consequently, thoracic impedance is not recommended for research applications. The American Academy of Sleep Medicine (AASM) recommends the use of an airflow sensor (e.g., oronasal thermistor, or nasal pressure) to measure respiration and detect apnea [10]. However, airflow measurements require that sensors be attached to the face. These sensors are poorly tolerated by infants during recovery from surgery as they interfere with both sleep and feeding.

The AASM guidelines also designate the respiratory inductive plethysmograph (RIP) as an alternative sensor for apnea detection [10]. RIP uses two elastic bands that encircle the torso to measure ribcage (RCG) and abdominal (ABD) respiratory movements. These bands are well tolerated by infants and do not interfere with clinical care or the infant’s behavioral state. RIP is the standard sensor for respiratory effort [10] in polysomnography and cardiorespiratory studies. It is also used to study respiration in other research applications including: prediction of extubation success in mechanically ventilated infants [11, 12], study of sudden infant death syndrome [13], and investigations of asthma [14] and bronchopulmonary dysplasia [15]. We have developed a data acquisition system that incorporates RIP sensors to monitor respiration, and a digital pulse oximeter to measure blood oxygen saturation (SAT) and photoplethysmography (PPG) [16], for the study of respiratory behavior of infants at risk of POA.

The investigation of POA using these data requires a consistent, reliable analysis method that fully characterizes the respiratory behavior of infants. The AASM endorses manual scoring as the “gold standard” for the study of apnea, and has published a set of rules to standardize the manual detection of apneas using RIP signals [10]. However these rules have 4 important limitations. First, they assume that the RIP signals are calibrated; that is, the RCG and ABD signals are scaled so that their sum is proportional to tidal volume. This process is valid for a fixed spinal angle and constant posture [17], but becomes inaccurate when the measurement conditions and/or breathing patterns change [18, 19]. Consequently, the RIP calibration is likely to change throughout a long recording session invalidating the accuracy of the calibrated sum, making its use questionable. Second, the AASM rules only define clinically relevant apnea events, but do not define other respiratory patterns such as short respiratory pauses, thoraco-abdominal asynchrony, sighs, and normal breathing. Yet, these other patterns are relevant to the comprehensive study of respiratory behavior, since there is evidence that POAs are associated with abnormal respiratory patterns [2]. Indeed, we have found that an increased frequency of respiratory pauses, longer than 2 s, was associated with POA [20]. Third, the AASM rules must be applied by certified sleep laboratory technicians. As a result the analysis is costly and not widely available, since many sleep laboratories have long waiting times [21]. This severely constrains the amount of data that can be analyzed. Fourth, even when the AASM rules are applied by certified sleep laboratory technicians, the results have low intra- and inter-operator repeatability [22]. This adversely affects studies where multiple scorers are needed (e.g., large datasets, longitudinal, multicenter), because the repeatability of the analysis decreases with the number of scorers.

Advancement of the study of POA requires that these limitations be addressed. To do so we believe it is necessary to: (i) adapt the manual scoring rules to analyze uncalibrated RIP data; (ii) define a comprehensive set of RIP patterns; (iii) provide a computer-aided, scoring tool to improve accuracy and consistency, and reduce the time required for manual analysis; and (iv) develop a training and evaluation strategy to standardize the analysis and improve intra- and inter-operator repeatability. This paper describes a comprehensive set of tools developed to address these needs. These tools comprise 5 components: (i) a clear, comprehensive set of definitions and scoring rules for 6 mutually exclusive RIP patterns, (ii) a computer aided tool for efficient manual scoring, (iii) a library of data segments representing each of the 6 RIP patterns, (iv) a formal training protocol for scorers to standardize performance, and (v) a method to monitor the ongoing performance of scorers.

This paper is organized as follows. Section II describes the 5 manual scoring tools introduced above. Section III describes the methods used to evaluate these tools. Section IV reports the results obtained by applying the tools to representative data from infants recovering from anesthesia. These results demonstrate that use of our tools produces efficient and accurate scoring with high intra- and inter-scorer repeatability regardless of operator expertise. Section V discusses the findings, and Section VI provides concluding remarks.

## Tools for Manual Scoring

### Pattern Definitions and Scoring Rules

Our objective was to define a comprehensive set of respiratory inductive plethysmography (RIP) patterns that would provide a complete description of the respiratory behavior on a continuous, sample-by-sample basis. To this end, we carried out an extensive literature review related to the scoring rules for infant RIP data. Key sources included: (i) the Infant Sleep Apnea section of the revised International Classification of Sleep Disorders: Diagnostic and Coding Manual from the American Academy of Sleep Medicine (AASM) [6]; (ii) the updated AASM Manual for the Scoring of Sleep and Associated Events: Rules, Terminology and Technical Specifications [10]; (iii) a series of articles on manual scoring published in the Journal of Clinical Sleep Medicine [23–30]; (iv) publications on POA in infants [1–4, 31]; and (v) publications on thoraco-abdominal synchrony in infants [15, 32, 33]. This led us to define 6, mutually exclusive, unique patterns that would comprehensively characterize RIP signals. These patterns are: synchronous-breathing (SYB), asynchronous-breathing (ASB), sigh (SIH), respiratory pause (PAU), movement artifact (MVT), and unknown (UNK). Table 1 describes each pattern in detail, and provides the scoring rules for the unambiguous assignment of each data sample to one of the 6 patterns.

**Table 1:** Unique, mutually-exclusive patterns of respiratory inductive plethysmography and their scoring rules.

Pattern	Definition	Scoring Rule	Example
Synchronous-breathing (SYB)	Quasi-sinusoidal breathing patterns in RCG and ABD, where the inspiration and expiration movements of RCG and ABD are in phase.	Phase difference of less than 90°.	Fig 3
Asynchronous-breathing (ASB)	Quasi-sinusoidal breathing patterns in RCG and ABD, where the RCG and ABD movements are out of phase.	Phase difference of 90° or more.	Fig 4
Sigh (SIH)	A breath with considerably larger amplitude and duration than preceding breaths.	Breath amplitude and duration twice that of the epoch’s average breath in both RCG and ABD.	Fig 5
Movement artifact (MVT)	A period during which both RCG and ABD signals are corrupted by movements not related to respiration.	RCG and ABD display a chaotic, non-sinusoidal, low frequency motion.	Fig 6
Respiratory pause (PAU)	A period where respiratory movements are absent in both RCG and ABD.	RCG and ABD have amplitudes less that 10% of those of the preceding normal breath. A PAU begins at the start of inspiration of the first breath that is clearly reduced, and ends with the start of inspiration of the first breath whose amplitude returns to the epoch’s average breath amplitude. If the start or end time of a PAU differs between RCG and ABD, the priority is given to the signal with higher SNR. All respiratory pauses are scored regardless of duration. Special cases:	Fig 1
		(i) PAU following SIH: RCG and ABD have amplitudes of less than 10% of that of the breath preceding the sigh in both signals.	Fig 7
		(ii) PAU following MVT: RCG and ABD have amplitudes of less than 10% of that of the breath that follows the pause in both signals.	Fig 8
Unknown (UNK)	Any other pattern arising from technical problems (e.g., loss of a connector, high noise), or ambiguous patterns (e.g., MVT during SYB, different patterns in RCG and ABD).	RCG and/or ABD do not correspond to any other pattern.	Fig 9

RCG = ribcage, ABD = abdomen, SNR = signal-to-noise ratio.

### RIPScore

RIPScore is an interactive computer application with a graphical user interface developed to support the efficient, manual scoring of RIP signals on a sample-by-sample basis. RIPScore is a redesign, and re-engineering of a rudimentary, prototype, manual scoring interface described in [34].

#### Main Screen

**Fig 1:**
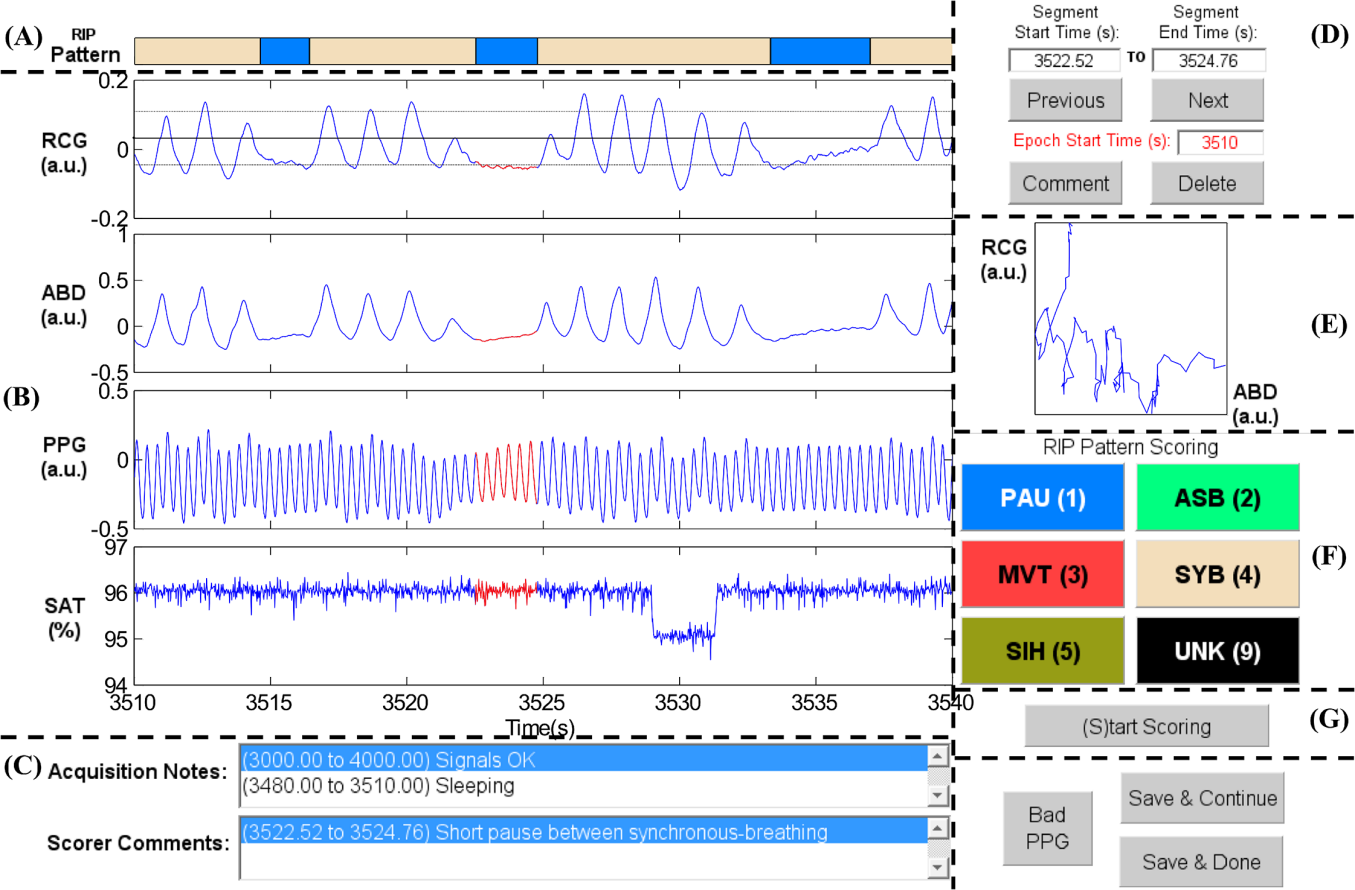
Elements of the RIPScore interface. (A) Respiratory Inductive Plethysmography (RIP) Pattern; (B) Signals from ribcage (RCG), abdomen (ABD), photoplethysmograph (PPG), and blood oxygen saturation (SAT); (C) Notes; (D) Segment and Epoch Control; (E) Lissajous Figure; (F) RIP Pattern Scoring; and (G) Mode Control. The epoch shows a representative example of Pause (PAU). The quasi-sinusoidal pattern in RCG and ABD stops during the PAU highlighted in red. The horizontal dotted cursors in RCG show an estimated variation of ± 90% of the amplitude of the breath preceding the PAU. Note that these cursors do not take into account low frequency trends, and so are only an approximate reference. a.u. = arbitrary units. *(A) RIP Pattern*: a color-coded bar showing the RIP pattern assigned by the scorer at each time; *(B) Signals*: plots of the cardiorespiratory signals including ribcage (RCG), abdomen (ABD), photoplethysmograph (PPG), and blood oxygen saturation (SAT). Clicking on a breath from RCG or ABD plots three horizontal cursors, one at the estimated breath’s amplitude, and two at ± 90% of that amplitude. Note that these cursors are not an exact amplitude reference for the epoch because they do not take into account low frequency trends frequently observed in RIP signals [35]; *(C)Notes*: text boxes showing time stamped notes made during data acquisition, and comments entered by the scorer during analysis; *(D) Segment and Epoch Control*: text boxes showing the start and end times for the current segment (highlighted in red in *Signals*); command buttons to add a “Comment” or “Delete” the RIP pattern assigned to the current segment; command buttons to scroll through epochs (“Previous”, “Next”), and a text box with the start time of the current epoch; *(E) Lissajous Figure*: a plot of RCG versus ABD for the current segment to aid the user in evaluating thoraco-abdominal synchrony. During breathing, the plot will be an ellipse tilted to the right for a phase less than 90 degrees, a circle for a phase of 90 degrees, and an ellipse tilted to the left for a phase greater than 90 degrees; *(F) RIP Pattern Scoring*: color-coded command buttons that assign a RIP pattern to the current segment; each button may also be activated by hitting the corresponding keyboard “hot-key” defined by the character in parenthesis for each button (e.g., the hot-key for Pause is ‘1’); *(G) Mode Control*: command button to switch between scoring and visualization mode.

RIPScore displays data in 30 s epochs, and allows the scorer to segment the signals and assign a RIP pattern to each segment. Fig 1 shows the main screen of RIPScore which comprises these main components:

#### Operating Modes

RIPScore has 4 operating modes: Visualization/Review, Scoring, Training, and Evaluation. These modes support different aspects of the scoring process.

Visualization/Review Mode supports viewing the signals and reviewing the RIP patterns and annotations assigned throughout the record. In this mode, the “Previous” and “Next” buttons scroll the data in 20 s increments. Entering a value in “Epoch Start Time” moves the epoch display to that value. The *RIP Pattern Scoring* buttons move the data to the next segment assigned to that pattern.

Clicking a segment on the *RIP Pattern* bar selects the segment, highlights the segment in *Signals*, plots the corresponding *Lissajous Figure*, and updates the segment start and end time text boxes. The “Comment” command can be used to assign a comment to the segment, while the “Delete” command removes the RIP pattern assigned to it.

Scoring Mode supports manual scoring. When activated, the cursor changes to crosshairs, the display moves to the first unscored segment, the segment start is set to the first unscored sample, and RIPScore prompts the user to select the end of the segment. The selected *Signals* segment is highlighted in red, and RCG and ABD are plotted in the *Lissajous Figure*. The scorer then assigns a RIP pattern to the segment using a *RIP Pattern Scoring* button or its hot-key; the segment’s assigned pattern, start and end time, and a timestamp are stored. The *RIP Pattern* bar is updated; and the display moves to the start of the next, unscored segment. This procedure continues until the scorer stops (by selecting the“(S)top Scoring” button) or all data have been scored. RIPScore then returns to Visualization/Review mode.

Training Mode supports the training of scorers by having users analyze simulated data with known RIP patterns. The interface is similar to that in Scoring Mode with the addition of an *Actual Pattern* bar for scored segments. If the trainee assigns an incorrect pattern to a segment, RIPScore displays an error message and provides the trainee with the opportunity to review the scored segment and reassign the pattern. Conversely, if the trainee assigns the correct pattern, RIPScore updates the *Actual Pattern* bar and allows the trainee to continue. A Training Mode session ends once the trainee has either: (i) scored the complete record, or (ii) correctly scored 5 patterns of each type consecutively.

**Fig 2:**
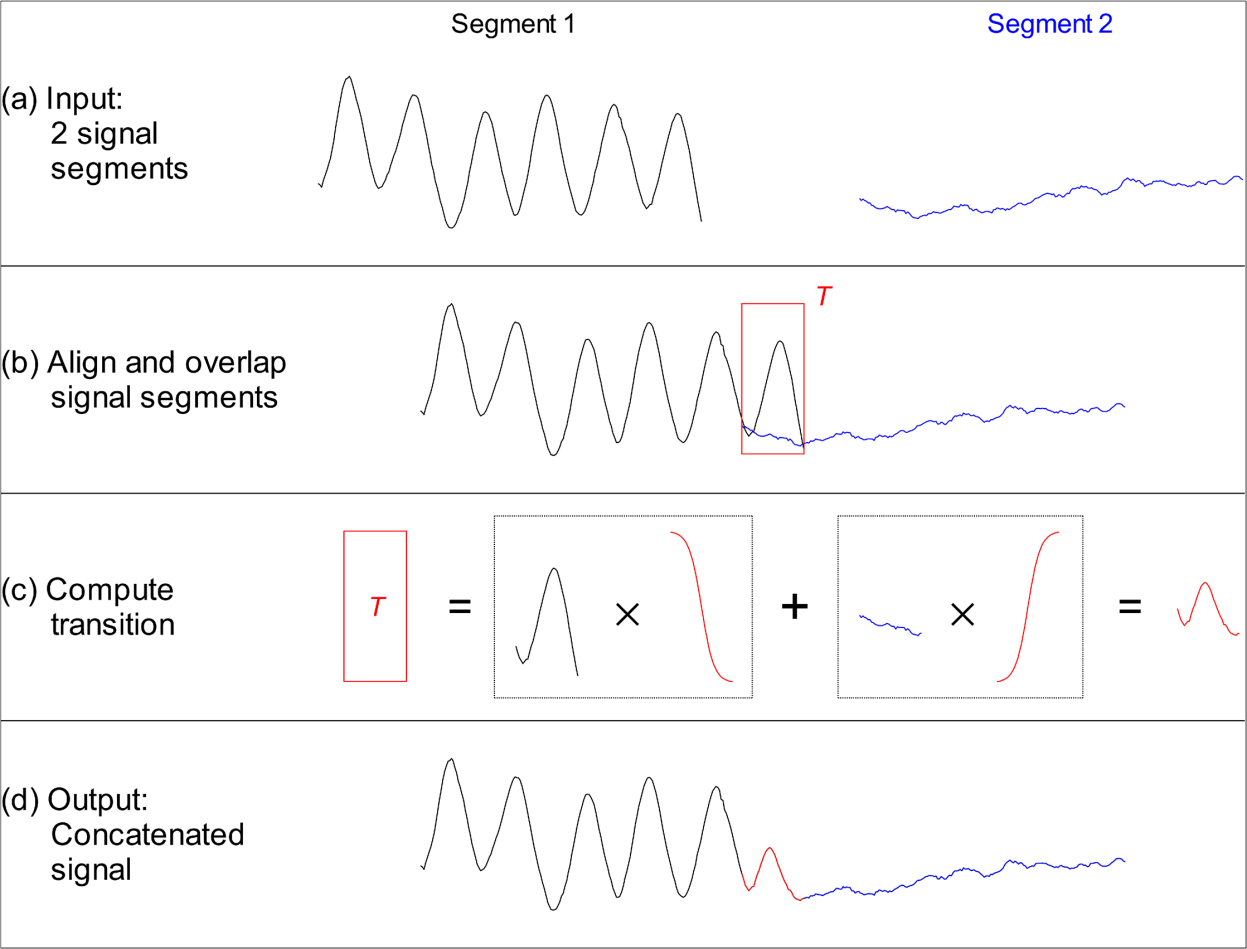
Concatenation of signal segments. (A) Sample input segments. (B) Input segments are aligned and overlapped over a transition window *T*. (C) The output during this window is computed by gradually attenuating the end of the first segment, gradually incrementing the start of the second segment, and adding the two parts to yield a smooth transition. (D) The output signal consists on the first segment up to the start of *T*, followed by the transition, followed by the second segment starting after *T*.

The simulated infant RIP records used in Training Mode are generated by concatenating, i.e., linking together, signal segments with known RIP patterns to yield continuous signals. Fig 2 illustrates the concatenation method, which consisted of the following 4 steps:

- two input signal segments were selected to be concatenated;
- the 2 signal segments were aligned with an overlap (transition window *T*) of *N*_*T*_ samples; that is, the last *N*_*T*_ samples of the first segment overlapped the first *N*_*T*_ samples of the second segment;
- the samples of the first segment in the transition window were gradually attenuated by multiplying them by a decaying sigmoid factor that varied from 1 to 0 over the length of the window; samples of the second segment were gradually amplified by multiplication with a sigmoid factor that increased from 0 to 1 over the window length; the modified signals in the transition window were then added to yield a smooth transition; and
- the output signal consisted on the first segment up to the start of *T*, followed by the transition, and then by the second segment starting after *T*.

The concatenation method overlapped the input segments to produce a smooth transition. This was done to avoid transition artifacts, which could generate sharp transients that do not resemble natural RIP patterns.

RIPScore uses two types of simulated data, and investigators are required to configure which type to use before scoring sessions start. Type I “simulated-pattern” data was based on signals generated using a breath-by-breath time-series model of infant breathing; other RIP patterns were simulated by manipulating these signals as described in [36]. Type II “true-pattern” data comprised segments of real data whose RIP pattern was determined during a reference analysis (REF) performed by one of the authors (KAB) as described below. Type II data were more complex and realistic than Type I because they incorporated the inherent variability of real infant breathing.

A new, 1 hr long, Training Mode data record is generated for each training session as follows:

- segments of each RIP pattern category are simulated and stored in a list, until the total length of data is > 1.5 hr;
- the list of simulated segments is re-ordered randomly;
- the list is examined to ensure that contiguous segments have different RIP patterns, if two contiguous segments have the same pattern, the second segment is pushed to the end of the list;
- the list is truncated to the first *N* segments whose total length is 1 hr; and
- the segments on the list are concatenated as described in Fig 2.

Evaluation Mode is used to evaluate a scorer’s accuracy and consistency. In this mode, the user analyzes a simulated data record with an interface similar to Training Mode, but with no feedback. Upon completion, RIPScore: (i) estimates the accuracy and consistency of the scorer; (ii) stores the accuracy and consistency values, the simulated data record, and the assigned RIP patterns; (iii) displays the accuracy and consistency to the scorer; and (iv) reveals the *Actual Pattern* bar in Review Mode so that the scorer can compare their assigned patterns to the actual, simulated patterns.

Data for Evaluation Mode are generated as follows:

- the first 30 min of data segments are simulated and stored in a list as for the Training data;
- the list is duplicated;
- the duplicate list is re-ordered randomly, and contiguous segments with equal RIP patterns pushed to the end;
- the two lists are joined, and the segments concatenated.

Thus, in the evaluation data record each simulated segment appears in both the first and second half but in a different, random order.

Performance is assessed in terms of the accuracy and consistency of the assigned RIP patterns. Accuracy is measured as the agreement between patterns assigned by the trainee and the actual pattern. Consistency is measured as the agreement between the patterns assigned to the same segments in the first and second half of the evaluation record. Agreement is quantified using the Fleiss’ kappa (*κ*) statistic [37, 38] computed on a sample-by-sample basis as in [36, 39]. This kappa implementation generalizes the traditional Cohen’s *κ* statistic [40] to evaluate agreement between multiple scorers when classifying observations into two or more categories.

**Sample Patterns in RIPScore.** Examples of the RIP patterns and special cases defined in Table 1 are illustrated in the following figures.

**Fig 3:**
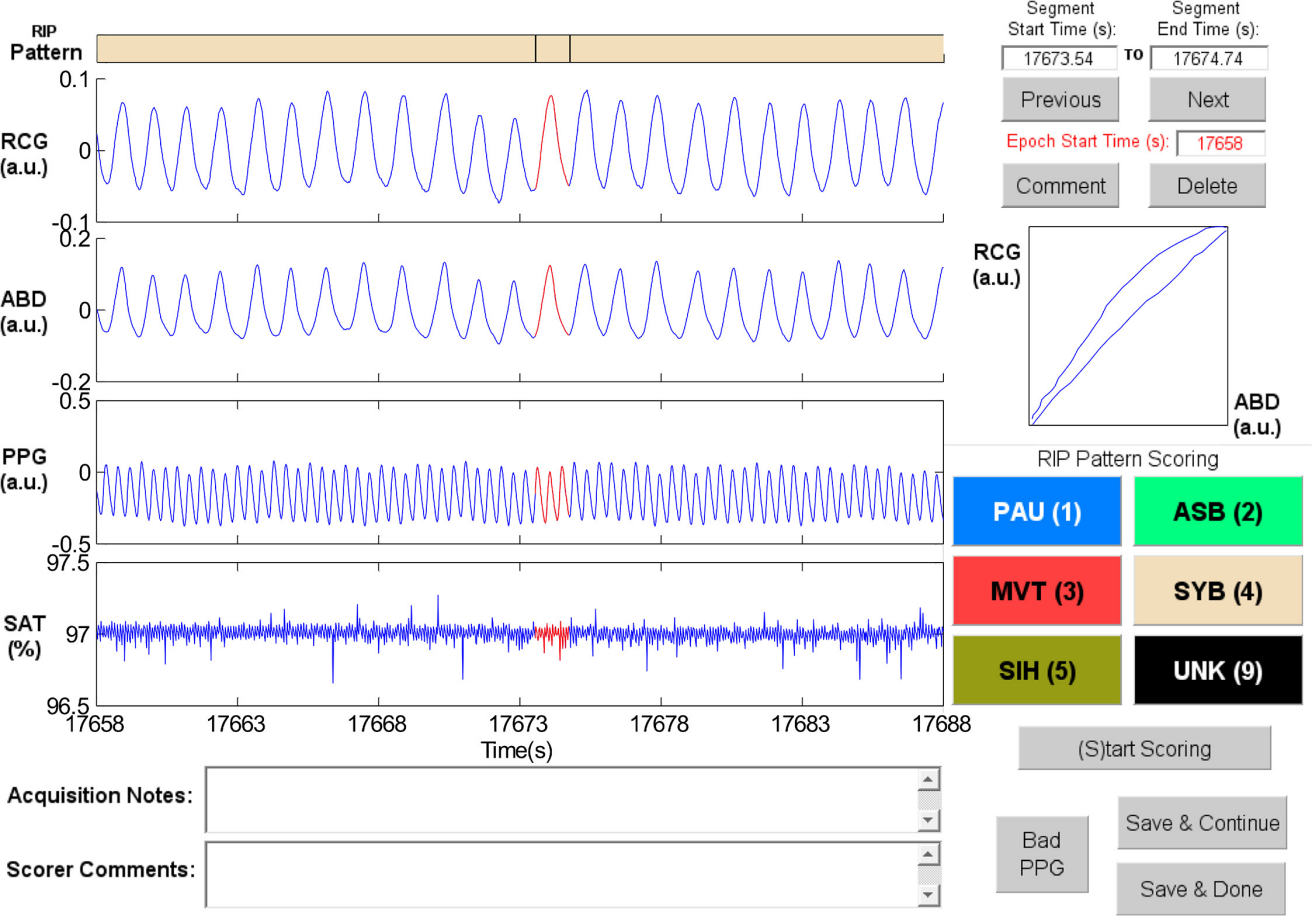
Representative example of Synchronous-Breathing (SYB). The ellipse in the Lissajous plot of ribcage (RCG) against abdomen (ABD) is tilted to the right. PPG = photoplethysmograph, SAT = blood oxygen saturation, a.u. = arbitrary units.

**Fig 4:**
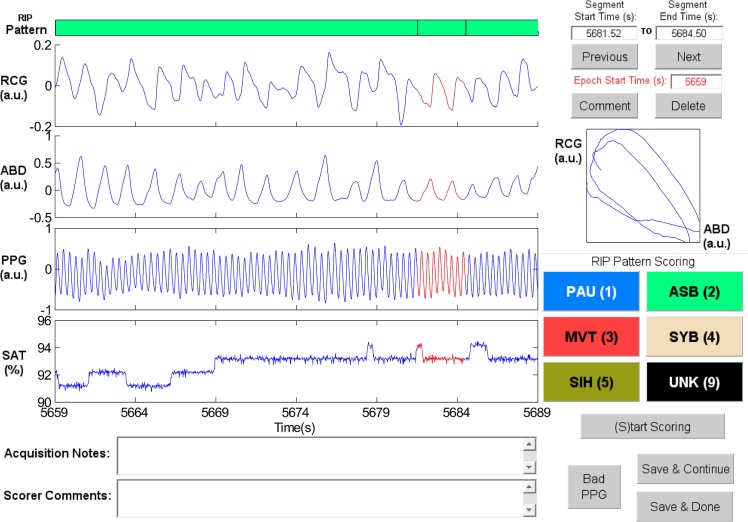
Representative example of Asynchronous-Breathing (ASB). The Lissajous plot of ribcage (RCG) against abdomen (ABD) for the segment highlighted in red shows ellipses tilted to the left. PPG = photoplethysmograph, SAT = blood oxygen saturation, a.u. = arbitrary units.

**Fig 5:**
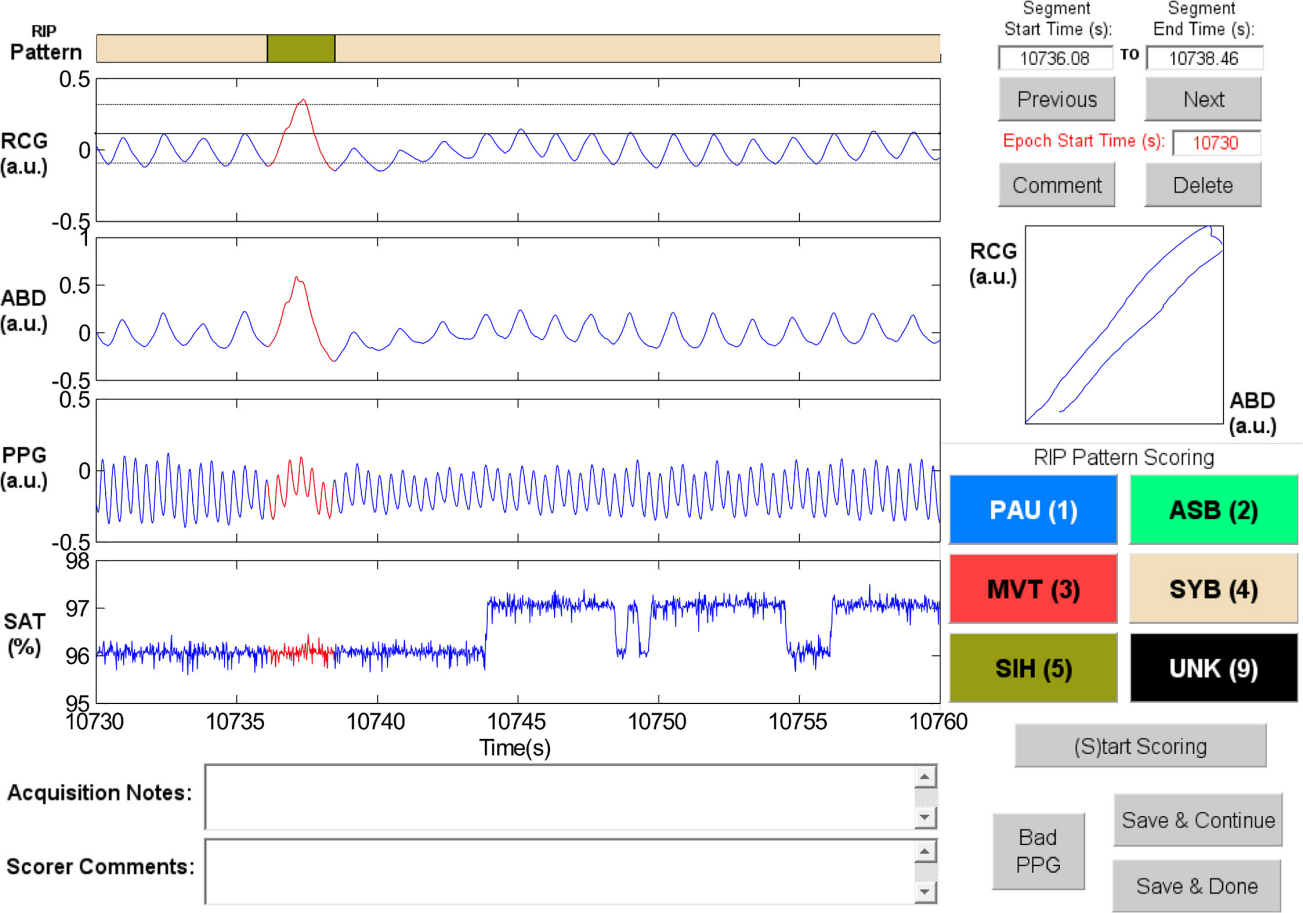
Representative example of Sigh (SIH). The SIH highlighted in red has larger amplitude and longer duration than the other breaths. The horizontal dotted cursors in the ribcage (RCG) signal show an estimated variation of ± 90% of the amplitude of the breath preceding the SIH. Note that these cursors are not an exact amplitude reference. Also, the Lissajous plot shows an ellipse tilted to the right. ABD = abdomen, PPG = photoplethysmograph, SAT = blood oxygen saturation, a.u. = arbitrary units.

**Fig 6:**
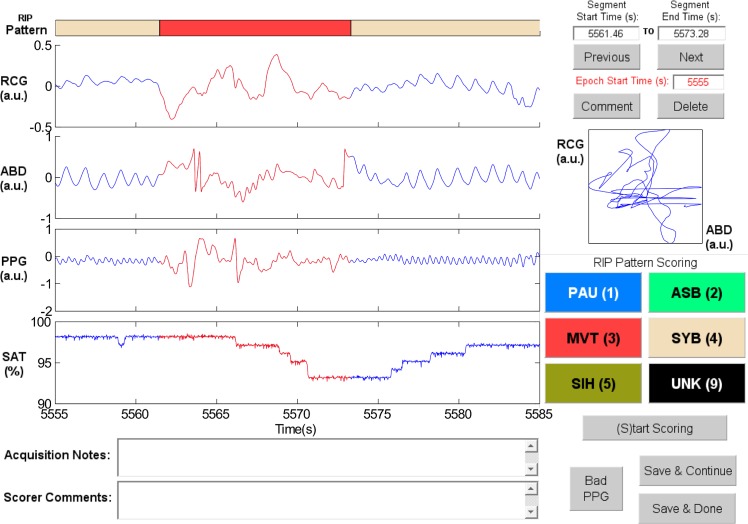
Representative example of Movement Artifact (MVT). The MVT in the ribcage (RCG) and abdomen (ABD) signals is highlighted in red. PPG = photoplethysmograph, SAT = blood oxygen saturation, a.u. = arbitrary units.

**Fig 7:**
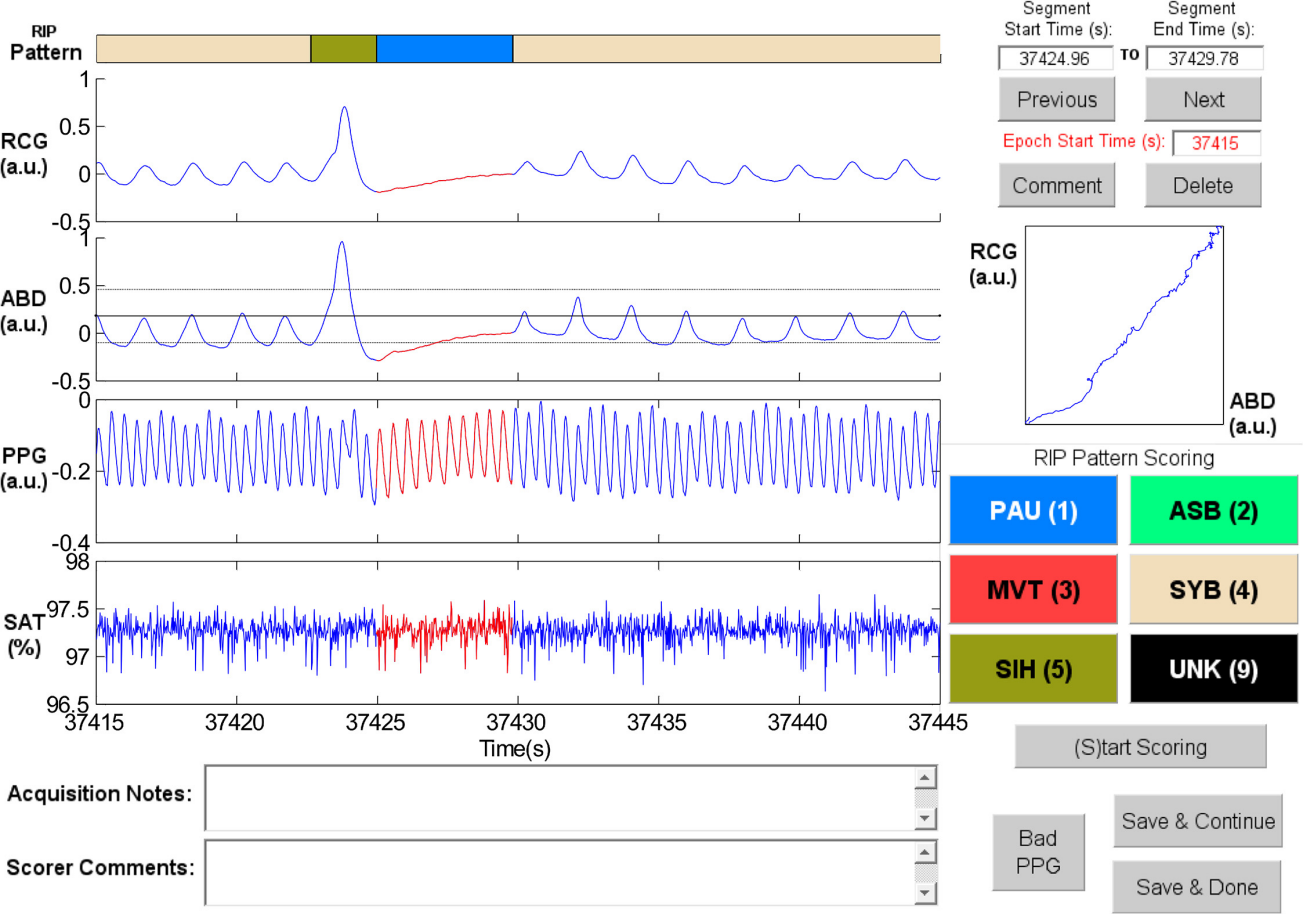
Representative example of a Pause (PAU) which follows a Sigh (SIH). The horizontal dotted cursors in the abdomen (ABD) signal show an estimated variation of ± 90% of the amplitude of the breath that precedes the SIH. Note that these cursors are not an exact amplitude reference. RCG = ribcage, PPG = photoplethysmograph, SAT = blood oxygen saturation, a.u. = arbitrary units.

**Fig 8:**
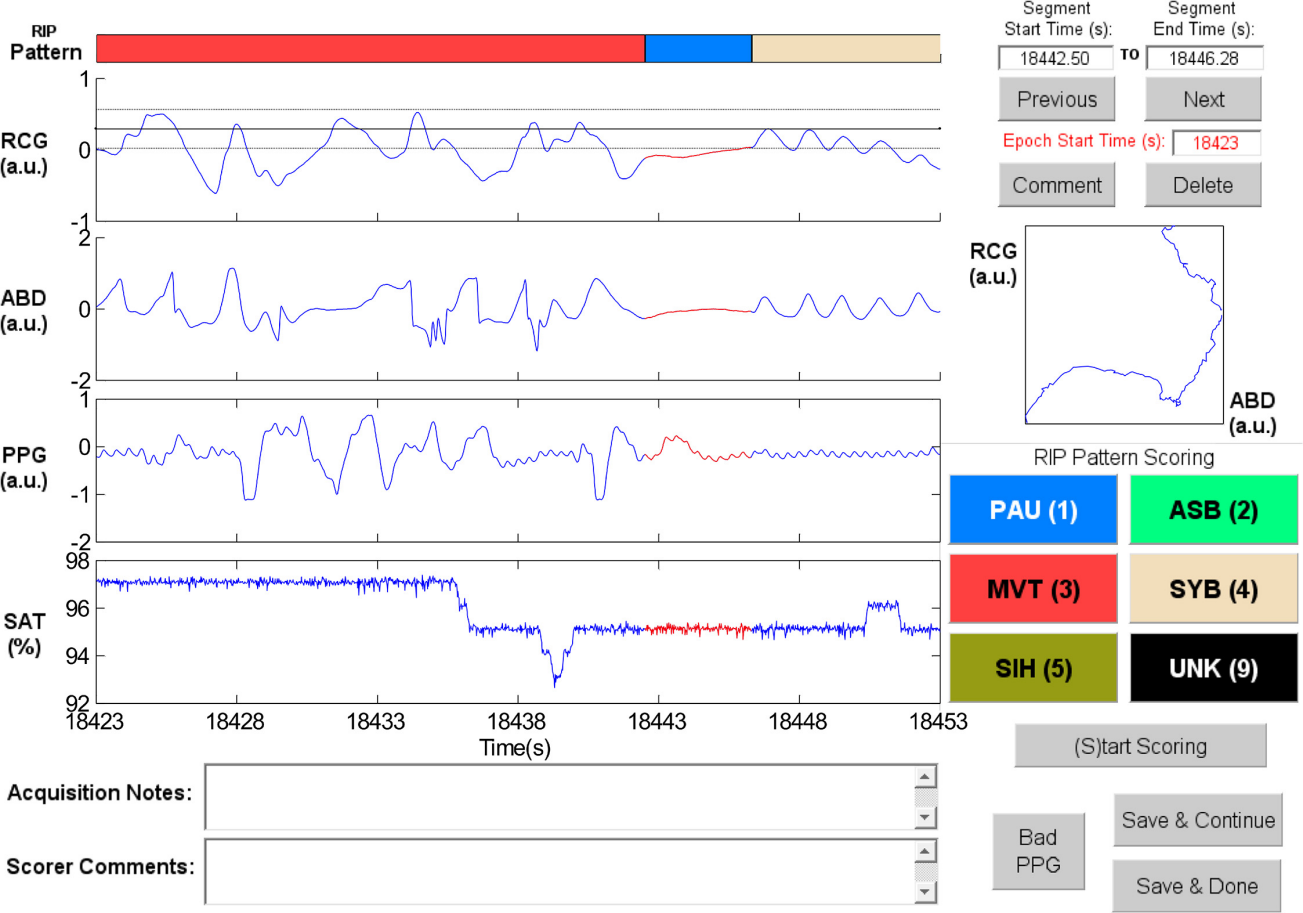
Representative example of a Pause (PAU) which follows a Movement Artifact (MVT). The horizontal dotted cursors in the ribcage (RCG) signal show an estimated variation of ± 90% of the amplitude of the breath that follows the PAU. Note that these cursors are not an exact amplitude reference. ABD = abdomen, PPG = photoplethysmograph, SAT = blood oxygen saturation, a.u. = arbitrary units.

**Fig 9:**
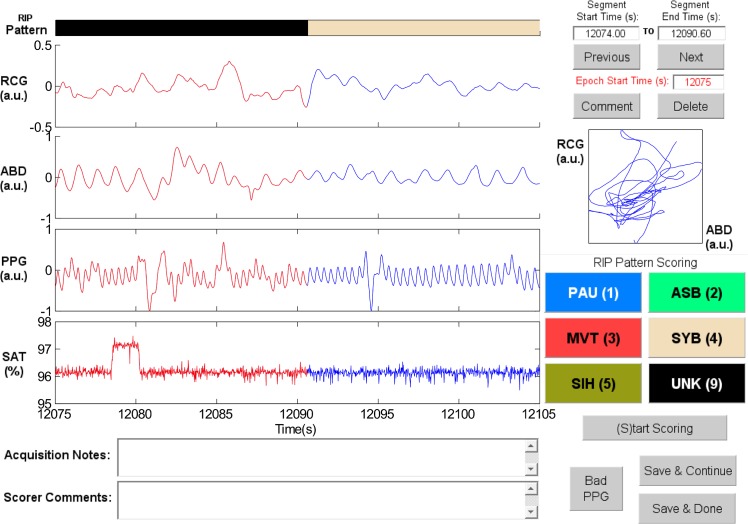
Example of Unknown (UNK). It is not possible to determine the pattern in the selected segment (red) because the ribcage (RCG) signal shows a low-frequency, chaotic pattern, while the abdomen (ABD) signal has a quasi-sinusoidal breathing pattern with an additional low-frequency movement component. PPG = photoplethysmograph, SAT = blood oxygen saturation, a.u. = arbitrary units.

- Synchronous-Breathing (SYB, Fig 3): the selected breaths in RCG and ABD (in red) are in phase, and the Lissajous plot is an ellipse tilted to the right;
- Asynchronous-Breathing (ASB, Fig 4): the selected breaths are out of phase, and the Lissajous plot is elliptical and tilted to the left;
- Sigh (SIH, Fig 5): the dotted horizontal cursor in RCG provides an approximate reference showing that the sigh has an amplitude of more than 190% of that of the preceding breath, with a duration longer than that of the other breaths;
- Movement Artifact (MVT, Fig 6): low-frequency motion corrupts both RCG and ABD;
- Pause (PAU, Fig 1): the pause at the middle of the epoch has an amplitude of less that 10% of that of the preceding breath, as evidenced by the horizontal cursor in RCG;
- PAU which follows a SIH (Fig 7): the horizontal cursors in the ABD signal show approximate reference amplitudes for the breath preceding the sigh; it is clear that the sigh’s amplitude is much larger, and that at least part of the pause’s amplitude is below the 10% dotted line;
- PAU which follows a MVT (Fig 8): the horizontal cursor in RCG suggests that the amplitude during the pause is less than 10% of that of the breath that follows the pause;
- Unknown (UNK, Fig 9): RCG and ABD have different patterns; RCG shows low-frequency movement artifact, while ABD shows breathing.

### Library of Segments with Known Patterns

A library containing “true-pattern” data segments representative of each of the 6 RIP patterns was created for use in RIPScore Training and Evaluation Modes.

#### Infant Data

The library was built using data acquired from 24 infants (19 male, birth age 31 ± 4 weeks, postmenstrual age 43 ± 2 weeks, weight 3.7 ± 1.0 kg) recruited for a prospective POA study. Inclusion criteria were: (i) postmenstrual age < 60 weeks at the time of surgery in preterm infants, and < 48 weeks in term infants, (ii) elective surgery for inguinal herniorrhaphy, and (iii) American Society of Anesthesiology physical status 1 or 2. Exclusion Criteria were: (i) post-operative admission to the Neonatal Intensive Care Unit or Pediatric Intensive Care Unit, (ii) emergency surgery, and (iii) spinal anesthesia. The anesthetic technique was not standardized.

Data were acquired in the Postanesthesia Care Unit (PACU) of the Montreal Children’s Hospital using a custom-built monitoring system [16]. Upon admission to the PACU, infant respibands (Inductobands, Ambulatory Monitoring Inc., Ardsley, NY, USA) were placed around the ribcage (at the nipple line) and abdomen (at the umbilicus) and interfaced with a Respiratory Inductive Plethysmograph (Battery Operated Inductotrace, Ambulatory Monitoring Inc., Ardsley, NY, USA). An infant oximeter probe (Nonin 8600 Portable Digital Pulse Oximeter, Nonin Medical Inc., Plymouth, MN, USA) was taped to a digit. The outputs were low-pass filtered (cut-off frequency 10 Hz) with an 8-pole, anti-aliasing, Bessel filter (Kemo, Jacksonville, FL, USA), sampled at 50 Hz, and stored. Subsequent, off-line analysis was performed using MATLAB (The MathWorks Inc., Natick, MA, USA). No attempt was made to calibrate the RIP signals. Recordings were 9.0 ± 2.2 hr long. Subsets of these data have been used in previous work [39, 41–43].

Recording sessions were continuously attended, and a paper record of the infant’s behavioral state, i.e., sleeping, feeding, diaper change, etc., was kept, referenced to the clock time and recording time. These handwritten entries were transcribed to an electronic text file and displayed as acquisition *Notes* in RIPScore. Demographic data and relevant clinical variables, including anesthetic and analgesic drug regimen, were recorded.

#### Ethics Statement

The study was approved by the Institutional Review Board of the McGill University Health Centre / Montreal Children’s Hospital (approval numbers PED-07-30, and 12-308-PED). Written, informed parental consent was obtained for each infant recruited to the study. Consent for publication of raw data was not requested specifically at the time the study was carried out. However, all materials have been thoroughly inspected, and all possible identifiers (as defined in [44]) were removed before the data were made available publicly. Thus, we believe that publication of these data poses negligible risk to the privacy of study participants.

#### Reference Manual Analysis

One of the authors (KAB) served as the reference scorer (REF). REF has extensive experience in the manual scoring of infant cardiorespiratory data, participated in the data acquisition, and contributed to the development of RIPScore.

REF used RIPScore to analyze the full records of 23 infants in two independent instances; the order in which the data records were analyzed was randomized between instances. One record was excluded because the infant was continuously handled by nurses and parents throughout the recording session. REF’s overall intra-scorer repeatability, measured with the Fleiss’ kappa statistic [37, 38], was “substantial” (*κ* = 0.80) [45]. Samples where REF assigned the same RIP pattern in the two instances were considered to be correct and defined the “true-pattern” for these samples.

**Fig 10:**
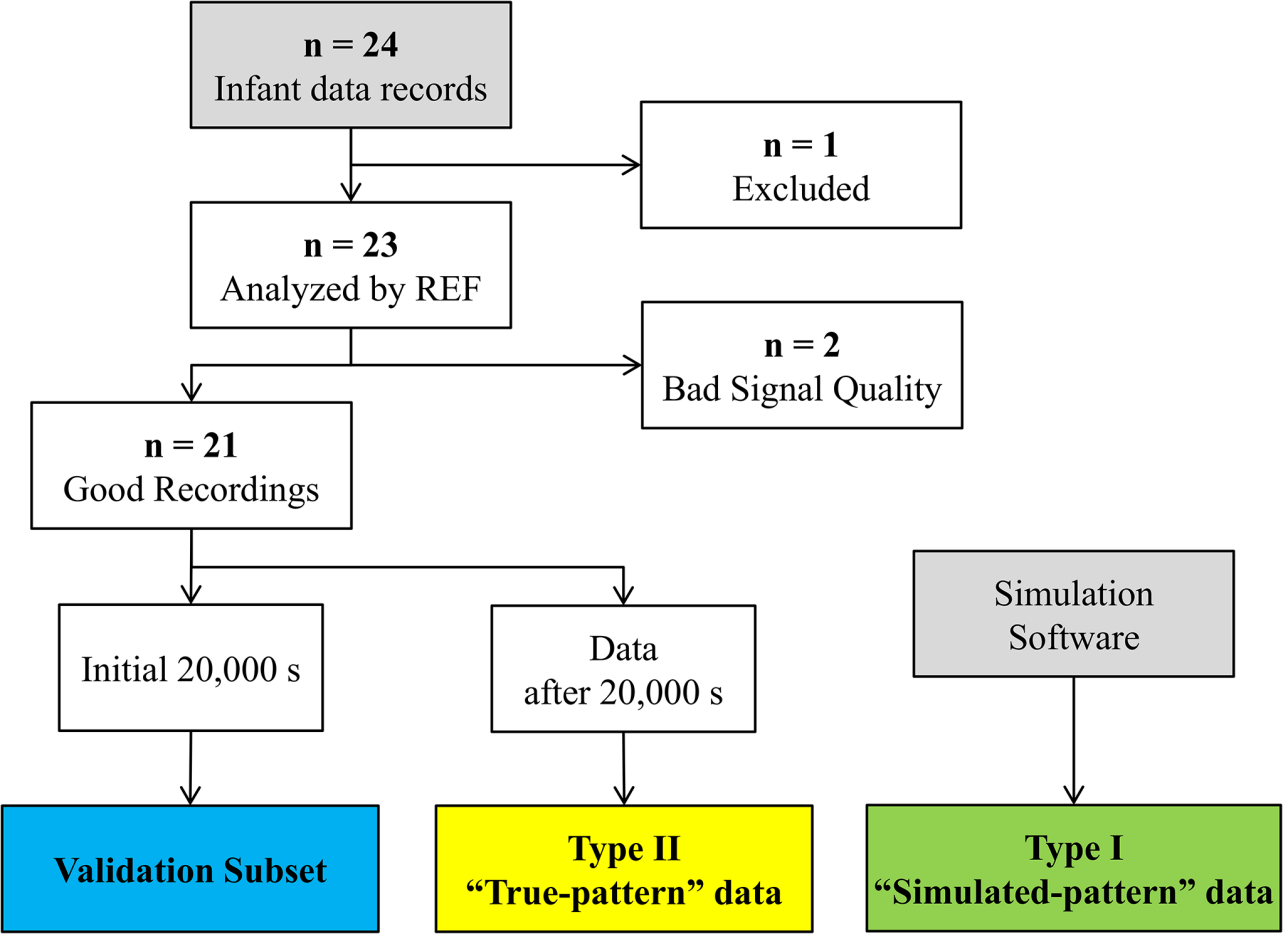
Study Data Flowchart.

This reference scoring task was very labor intensive and required 8 months to complete. For this reason, data were partitioned into two subsets: (i) a validation subset used to evaluate the performance of scorers, and (ii) a library of “true-pattern” segments used to generate the Type II “true-pattern” simulated data. Fig 10 summarizes how the validation subset and the “true-pattern” segment library were created.

The validation subset comprised data from 21 infants, truncated to a maximum of 20,000 s per record, representing a 54% of the complete data set. Records from 2 infants that were analyzed by REF were excluded due to bad quality in the recordings. To ensure that the validation subset was representative, the proportion of “true-pattern” samples assigned to each RIP pattern was computed for both the complete and truncated data records. The Wilcoxon signed rank test [46] indicated that the proportions were not significantly different as Table 2 shows.

**Table 2:** Proportion of “true-pattern” samples in the records used to create the validation data subset.

Pattern	Complete Record	Truncated, Validation Record	p-value
**SYB**	0.73 [0.08]	0.75 [0.06]	0.13
**ASB**	0.03 [0.05]	0.02 [0.05]	0.13
**SIH**	0.01 [0.00]	0.01 [0.00]	0.28
**PAU**	0.02 [0.03]	0.02 [0.02]	0.25
**MVT**	0.12 [0.03]	0.12 [0.06]	0.15
**UNK**	0.08 [0.04]	0.08 [0.04]	0.39

Results presented as median [interquartile range]. SYB = synchronous-breathing, ASB = asynchronous-breathing, SIH = sigh, PAU = respiratory pause, MVT = movement artifact, UNK = unknown. The library of “true-pattern” segments was created from remaining data and comprised 16,285 segments.

### Training Protocol

All scorers underwent a common training protocol, using RIPScore Training and Evaluation Modes, to standardize the analysis and performance of scorers using our tools.

**Fig 11:**
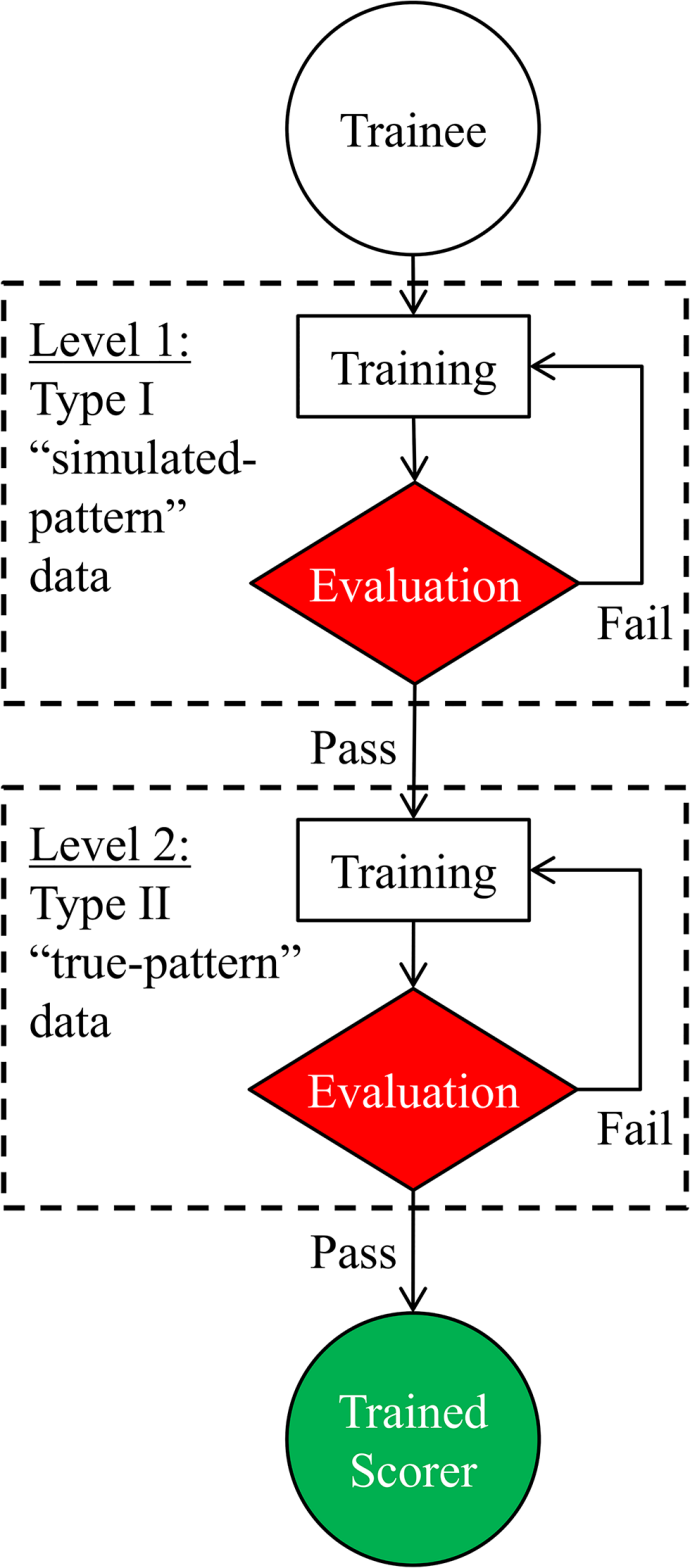
Scorer training protocol. Criteria to successfully complete levels: (A) Level 1, the trainee obtained accuracy and consistency values of *κ* ≥ 0.8; and (B) Level 2, the trainee obtained accuracy and consistency values of *κ* ≥ 0.8 on two consecutive sessions.

**Fig 12:**
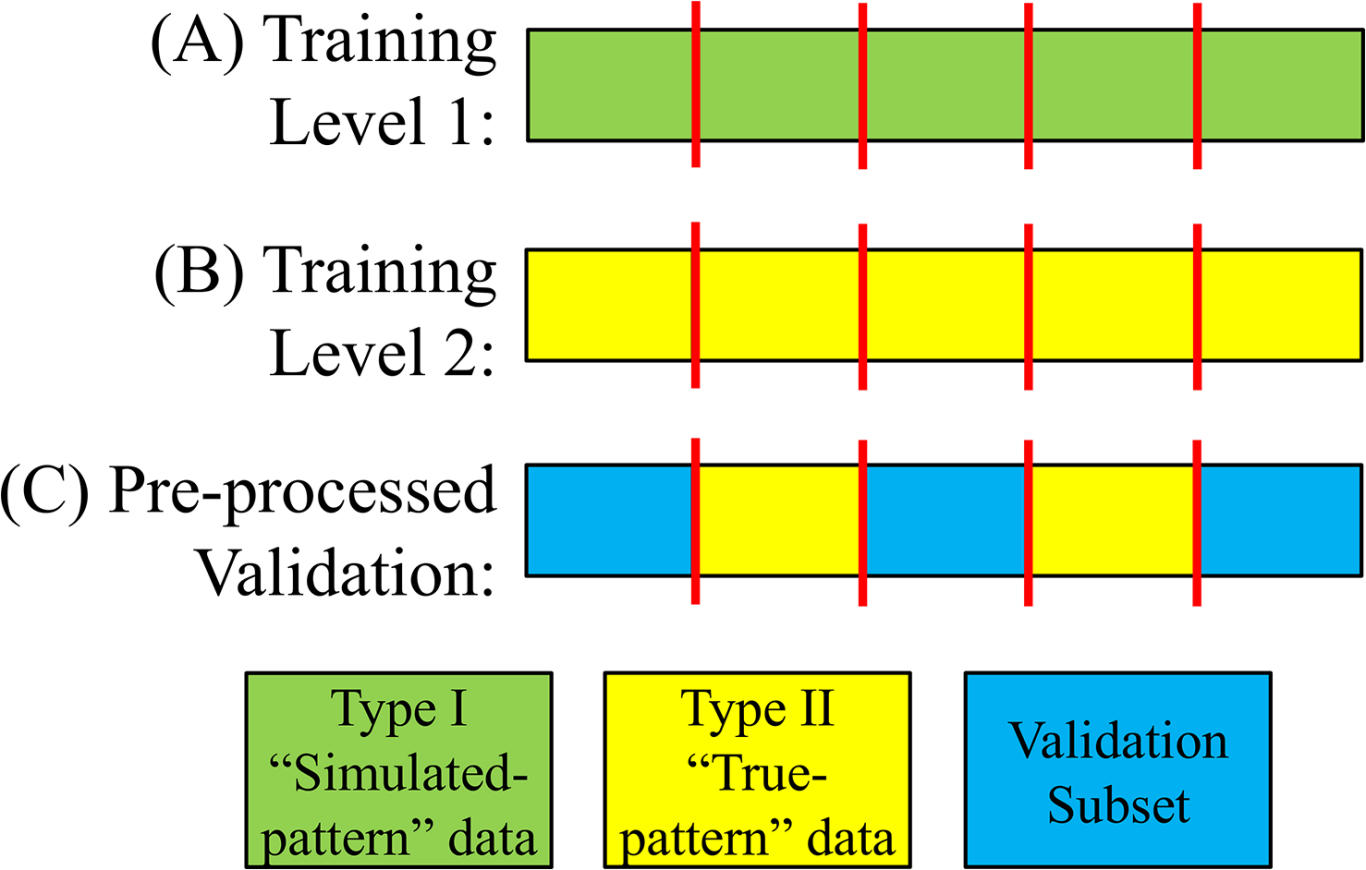
Data formats. (A) Type I, and (B) Type II data segments were concatenated to generate the training records. (C) Validation records were pre-processed such that Type II segments were inserted into the validation subset. Red vertical lines indicate the concatenation point.

Fig 11 shows a block diagram of the training protocol. Training had 2 levels, each having two stages: training and evaluation. Trainees started at Level 1, where they were familiarized with RIPScore, the 6 mutually exclusive RIP pattern definitions, and the scoring rules, by analyzing Type I “simulated-pattern” records (Fig 12A). Each level began with a training stage where trainees scored data in RIPScore Training Mode. Upon completing the training stage, their accuracy and consistency were evaluated using RIPScore Evaluation Mode. If their performance was adequate (see Fig 11) they advanced to Level 2 of training, if not, they repeated the Level 1 training stage.

Level 2 training proceeded in a similar manner except that the data analyzed were the more realistic Type II “true-pattern” data records (Fig 12B). Training was completed after successful completion of the Level 2 evaluation stage (see Fig 11).

Reference values of performance were obtained by having REF analyze two sessions of each training level. These analyses showed that REF had excellent consistency and accuracy values ranging from *κ* = 0.76 to *κ* = 0.89.

### Monitoring of Scorers for Quality Control

Scorer accuracy and consistency were evaluated on a record-by-record basis using a quality control method based on the pre-processing phase described next.

#### Pre-processing

The validation dataset was pre-processed by inserting Type II “true-pattern” segments into each data record (Fig 12C). Thus, for this pre-processing phase, a total of 152 segments (1,000 s worth of data) were selected from the “true-pattern” segment library, such that each RIP pattern was equally represented. The distribution of these 152 segments was: 25 SYB, 26 ASB, 27 SIH, 22 PAU, 27 MVT, and 25 UNK.

For each data record in the validation subset, the 152 segments were randomly ordered and inserted into the first 3 hrs of the record at randomly selected times. These “true-pattern” segments were then randomly re-ordered, and inserted into the last 3 hr of the record at random times. Segments were inserted by splitting the data record (see Fig 12C), and concatenating the segment as in Fig 2. Thus each of the 21 pre-processed data records contained two copies of the 152 “true-pattern” segments.

These inserted “true-pattern” segments were then used to evaluate scorer accuracy and consistency using the same methods as in RIPScore’s Evaluation Mode.

## Evaluation of the Manual Scoring Tools

The manual analysis tools were evaluated by examining the performance of three scorers in the analysis of the pre-processed validation data subset.

### Scorer Recruitment and Training

The three scorers had quite different backgrounds and experience in the analysis of respiratory data. The first (SC1) was a pediatric anesthesiologist with expertise in infant respiratory physiology, who participated in data acquisition and is a co-author (GB). The second (SC2) was a senior respiratory pediatric sleep laboratory technician with extensive experience in manual scoring of pediatric cardiorespiratory data. The third (SC3) was a computer network analyst with a master’s degree in telecommunications but no clinical expertise. All three scorers were trained using the protocol.

### Validation of the Manual Analysis Tools

The three scorers analyzed the entire, pre-processed, validation data subset in two independent, blinded instances; the order of the data records was randomized between instances and between scorers. Scorer performance was evaluated in terms of the following parameters.

#### Accuracy and Consistency

The two copies of the 152 “true-pattern” segments inserted in each data record were analyzed to evaluate the scorers’ ongoing accuracy and consistency.

#### Scoring Rate

The time required to score a data record was estimated by summing the difference between the timestamps of consecutive scores. Differences greater than 2 min were excluded because they likely resulted from interruptions in the analysis. The overall scoring rate was estimated as the ratio of the length of a data record (in data hours) to the hours required to score it. Pattern-specific scoring rates were estimated as the ratio of the total length of segments assigned to a RIP pattern to the time required to score those segments.

#### Intra and Inter-Scorer Repeatability

Intra- and inter-scorer repeatability of the RIP patterns assigned to the validation data were assessed using the Fleiss’ kappa (*κ*) statistic [37, 38] on a sample-by-sample basis.

#### Confusion Analysis

Confusion in the scoring of the 6 RIP patterns Θ = {*SYB*, *ASB*, *SIH*, *PAU*, *MVT*, *UNK*} was assessed by computing the confusion matrix **P** whose elements *P*_*i*,*j*_ gave the conditional probability that a sample with consensus pattern *i* would be scored as pattern *j*. A sample *x*_*k*_ was assigned a consensus RIP pattern *Cn*(*x*_*k*_) ∈ Θ if it was assigned that pattern in the absolute majority (4 or more) of the 6 scoring iterations. Samples without consensus pattern were excluded from the confusion analysis. Thus, to estimate *P*_*i*,*j*_ for each scorer, the *N*_*i*_ samples with consensus pattern *i* were identified. Then, *N*_*j*_, the number of times the *N*_*i*_ samples had been assigned to pattern *j*, was determined. Finally, the conditional probability was estimated as *P*_*i*,*j*_ = *N*_*j*_ / *N*_*i*_. Confusion matrices were computed for each scorer separately, and also as a group.

To assess the effects of segment length, confusion matrices were also computed after excluding scored segments shorter than a threshold (varied from 0 s to 20 s).

### Statistical Analysis

Bootstrapping [47] with 100 resamples was used to estimate the standard deviation of the *κ* values and the confusion matrix probabilities. Values of *κ* were interpreted according to the intervals proposed in [45]: *κ* < 0 = poor, 0 ≤ *κ* ≤ 0.2 = slight, 0.2 < *κ* ≤ 0.4 = fair, 0.4 < *κ* ≤ 0.6 = moderate, 0.6 < *κ* ≤ 0.8 = substantial, and 0.8 < *κ* ≤ 1 = almost perfect. Random selections were drawn from a uniform distribution where all instances had equal probability of being selected.

## Results

### Training

Tables 3 and 4 show the accuracy and consistency of the scorers for each training session and level. All scorers reached the required Level 1 performance (*κ* ≥ 0.8) after the first session. None of the scorers reached the required performance in the first Level 2 session; SC1 and SC3 had low accuracy, and SC1 and SC2 had low consistency. Scorer performance improved with training and all 3 achieved the required level of accuracy and consistency (*κ* ≥ 0.8) in sessions 2 and 3 of Level 2, completing the training protocol requirements.

**Table 3:** Training accuracy.

Scorer	Level 1	Level 2		
	*Session 1*	*Session 1*	*Session 2*	*Session 3*
**SC1**	0.94	0.72	0.82	0.81
**SC2**	0.94	0.81	0.86	0.87
**SC3**	0.94	0.79	0.82	0.81

Level 1 = Type I “simulated-pattern” data. Level 2 = Type 2 “true-pattern” data. Performance was measured using the Fleiss’ *κ* statistic [37]. The standard deviation was < 0.01 in all cases.

**Table 4:** Training consistency.

Scorer	Level 1	Level 2		
	*Session 1*	*Session 1*	*Session 2*	*Session 3*
**SC1**	0.89	0.74	0.86	0.81
**SC2**	0.90	0.76	0.83	0.84
**SC3**	0.93	0.86	0.85	0.80

Level 1 = Type I “simulated-pattern” data. Level 2 = Type 2 “true-pattern” data. Performance was measured using the Fleiss’ *κ* statistic [37]. The standard deviation was < 0.01 in all cases.

### Accuracy and Consistency

**Fig 13:**
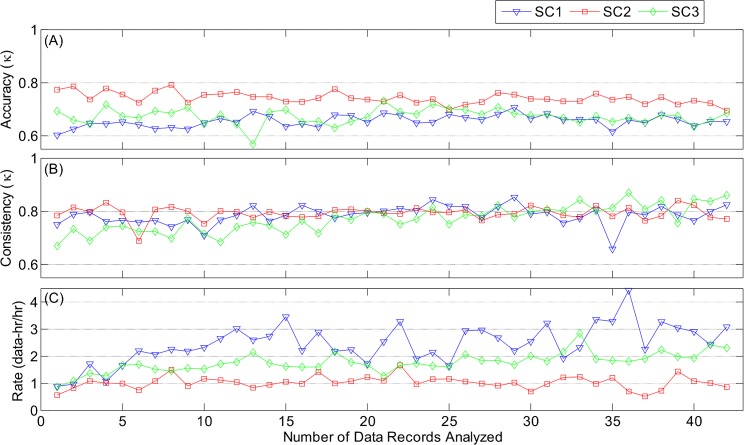
Overall scoring performance. (A) Accuracy (Fleiss’ *κ*); (B) consistency (Fleiss’ *κ*); and (C) rate (hours of data per hour of scoring) as a function of number of data records analyzed. SC1 was a pediatric anesthesiologist; SC2 was an experienced sleep laboratory scorer; and SC3 was a data networks analyst with no clinical experience. Standard deviation of each accuracy and consistency point was < 0.01.

Fig 13 documents the performance of the scorers as a function of the number of records scored. Fig 13A shows that the overall scoring accuracy was substantial and nearly constant throughout the scoring effort for all three scorers (SC1: *κ* = 0.66 ± 0.02, SC2: *κ* = 0.74 ± 0.02, SC3: *κ* = 0.67 ± 0.03). Consistency (Fig 13B) was high throughout for SC1 (*κ* = 0.79 ± 0.03) and SC2 (*κ* = 0.79 ± 0.02); SC3 (*κ* = 0.77 ± 0.05) started slightly lower, but quickly reached a level similar to the other scorers.

**Fig 14:**
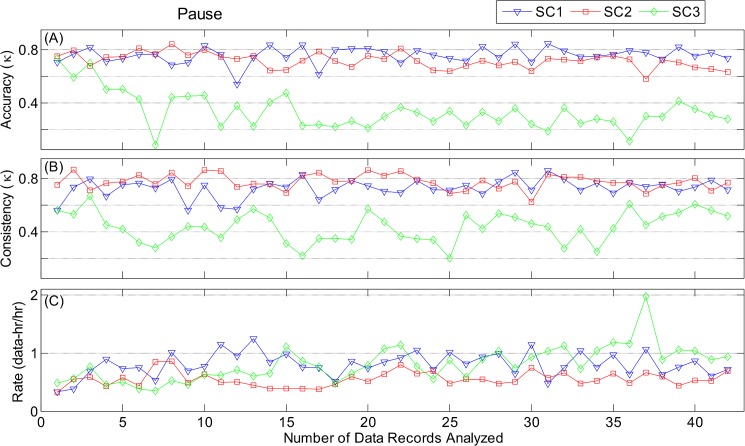
Evaluation of manual scoring of Pause. (A) Accuracy (Fleiss’ *κ*); (B) consistency (Fleiss’ *κ*); and (C) rate (hours of data per hour of scoring) as a function of number of data records analyzed. Results are shown for the 42 data records analyzed (21 files scored twice).

Analysis of pattern-specific accuracy and consistency revealed some substantial differences between scorers for 3 RIP patterns: PAU, MVT, and UNK. For PAU, Fig 14 shows that two scorers had high, nearly constant levels of accuracy (SC1: *κ* = 0.76 ± 0.06, SC2: *κ* = 0.72 ± 0.06) and consistency (SC1: *κ* = 0.73 ± 0.07, SC2: *κ* = 0.78 ± 0.06). In contrast, SC3, the scorer with non-clinical background, had lower accuracy (*κ* = 0.34 ± 0.14) and consistency (*κ* = 0.44 ± 0.11). For MVT (S1 Fig), the three scorers had similar consistency, but a range of accuracies, with SC2 having the highest (*κ* = 0.75 ± 0.03), followed by SC3 (*κ* = 0.65 ± 0.07), and SC1 with the lowest (*κ* = 0.53 ± 0.02). For UNK (S2 Fig), the accuracy of SC2 (*κ* = 0.54 ± 0.07) and SC3 (*κ* = 0.46 ± 0.06) were moderate, while that of SC1 was poor (*κ* = 0.03 ± 0.05). As would be expected the consistency of SC1 for UNK was much lower (*κ* = 0.29 ± 0.09) than those of SC3 (*κ* = 0.66 ± 0.11), and SC2 (*κ* = 0.58 ± 0.06).

The 3 scorers had similar accuracy and consistency for SYB, ASB, and SIH (S3–S5 Figs).

### Scoring Rate

Fig 13C demonstrates some significant differences in scoring rate among the scores. All three scorers began scoring at a rate of 1 data-hr/hr, but SC1 and SC3 gradually increased the scoring rate by two- to three-fold throughout the study. In contrast, SC2 maintained a constant rate throughout. Analysis of the pattern-specific rates showed that the increase in scoring rate was primarily associated with SYB (S3 Fig), and MVT (S1 Fig), while scoring rates for ASB (S4 Fig), SIH (S5 Fig), PAU (Fig 14), and UNK (S2 Fig) were fairly constant throughout.

### Repeatability

Each scorer analyzed the pre-processed validation subset in two independent, randomized instances. Intra-scorer repeatability was assessed by comparing the RIP patterns each scorer assigned to the same data in the two instances. Table 5 shows that the overall intra-scorer repeatability was very good; the scorer who participated in data acquisition SC1 had the highest repeatability (*κ* = 0.84), followed by the sleep laboratory technician SC2 (*κ* = 0.77), and the non-clinical scorer SC3 (*κ* = 0.72). The pattern with the highest intra-scorer repeatability was SYB (0.84 ≤ *κ* ≤ 0.89), and the pattern with the lowest intra-scorer repeatability was UNK (0.49 ≤ *κ* ≤ 0.56).

**Table 5:** Intra-scorer repeatability.

Scorer	Overall	SYB	ASB	SIH	PAU	MVT	UNK
**SC1**	0.84	0.89	0.78	0.73	0.79	0.88	0.49
**SC2**	0.77	0.86	0.79	0.58	0.78	0.76	0.56
**SC3**	0.72	0.84	0.70	0.67	0.74	0.64	0.53

Repeatability was measured using the Fleiss’ *κ* statistic [37]. Standard deviation was < 0.01 in all cases. SYB = synchronous-breathing, ASB = asynchronous-breathing, SIH = sigh, PAU = respiratory pause, MVT = movement artifact, UNK = unknown.

Inter-scorer repeatability was computed for each of the 8 unique analysis combinations (each combination comprised one analysis iteration from each of the 3 scorers, and each scorer performed 2 iterations). Table 6 reports the result as mean ± standard deviation. The overall inter-scorer repeatability was *κ* = 0.65. The RIP pattern with most repeatability was SYB (*κ* = 0.81), and the repeatability on PAU was substantial (*κ* = 0.65).

**Table 6:** Inter-scorer repeatability of scorers SC1, SC2, and SC3.

Overall	SYB	ASB	SIH	PAU	MVT	UNK
0.65 ± 0.02	0.81 ± 0.01	0.69 ± 0.01	0.53 ± 0.01	0.65 ± 0.02	0.58 ± 0.04	0.28 ± 0.03

Repeatability was measured using the Fleiss’ *κ* statistic [37]. Results are presented as mean ± standard deviation. SYB = synchronous-breathing, ASB = asynchronous-breathing, SIH = sigh, PAU = respiratory pause, MVT = movement artifact, UNK = unknown.

### Confusion Analysis

Table 7 presents the proportion of samples assigned to each consensus RIP pattern in the validation dataset. There was a consensus for 90% of the samples; with the most common pattern being SYB (65%), and the least frequent being SIH (1%). For completeness, we computed the pattern proportions for the remaining 10% of samples with no consensus even though these data were not used in the confusion analysis. We found that the majority (60%) of the non-consensus samples were scored as either UNK or MVT, and the rest were: SYB 22%, ASB 8%, SIH 3%, and PAU 7%. We later found that the proportion of samples without consensus pattern could be reduced to 5% if all samples scored as MVT were to be re-assigned to UNK.

**Table 7:** Proportion of consensus patterns for the confusion analysis.

Consensus Pattern	Number of Samples	Proportion
**SYB**	12,877,448	0.65
**ASB**	859,835	0.04
**SIH**	145,352	0.01
**PAU**	632,694	0.03
**MVT**	2,606,271	0.13
**UNK**	810,583	0.04
**None**	2,017,540	0.10

SYB = Synchronous-breathing, ASB = asynchronous-breathing, SIH = sigh, PAU = respiratory pause, MVT = movement artifact, UNK = unknown.

**Fig 15:**
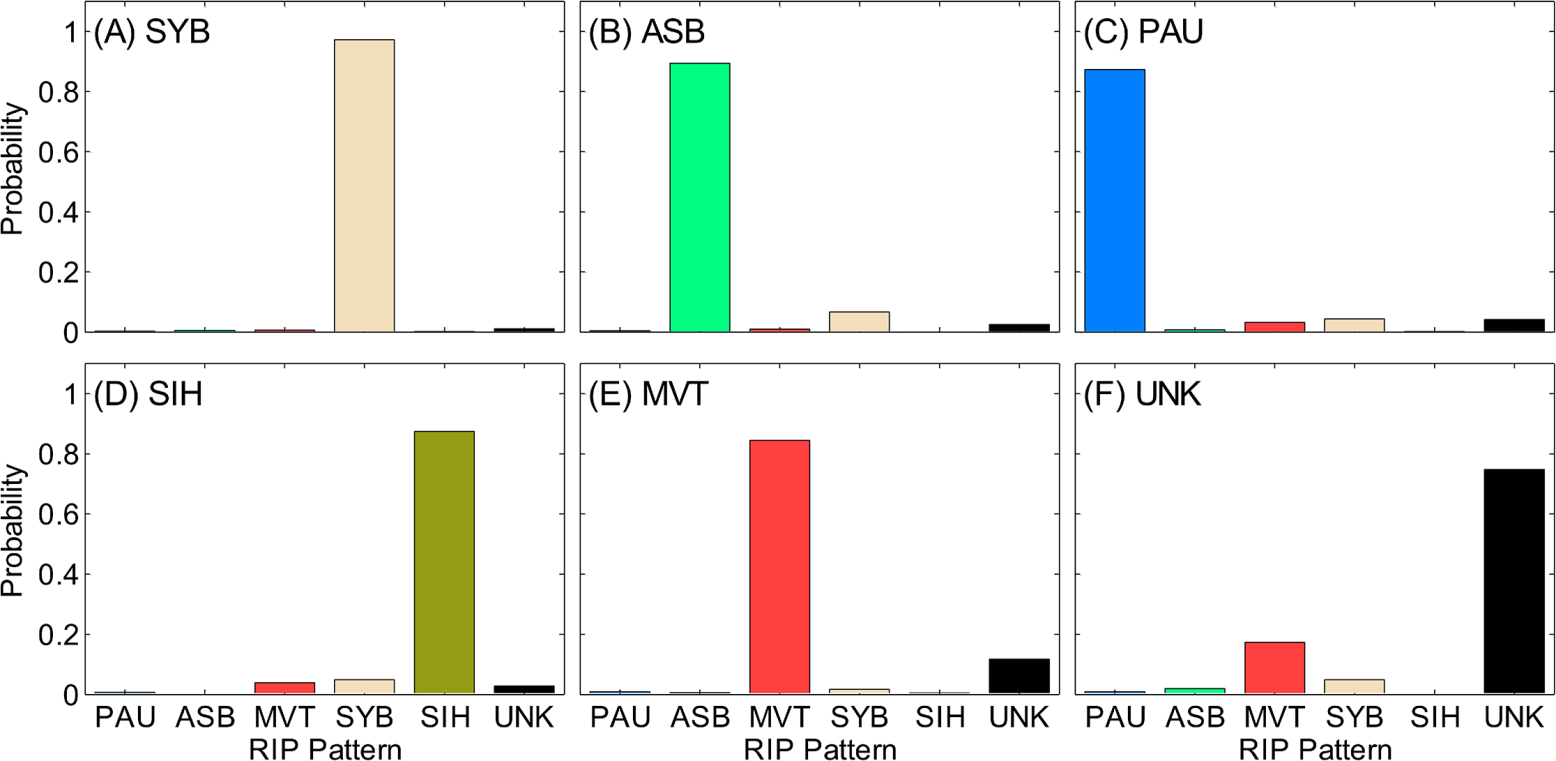
Confusion matrix. Conditional probability of each respiratory inductive plethysmography (RIP) pattern for samples with the consensus pattern of: (A) synchronous-breathing (SYB), (B) asynchronous-breathing (ASB), (C) pause (PAU), (D) sigh (SIH), (E) movement artifact (MVT), and (F) unknown (UNK). When there is no confusion, the consensus pattern has a probability of 1 and the others have probabilities of 0. During total confusion all patterns have equal probabilities. Standard deviations of all probabilities were < 0.01.

Fig 15 shows the confusion matrix for the full data set (3 scorers combined for all segment lengths). It is evident that there was no systematic confusion of samples with consensus pattern of SYB, ASB, PAU, or SIH. A significant confusion was evident between UNK and MVT (Fig 15F). The confusion matrices for the individual scorers showed similar results (see S6–S8 Figs).

**Fig 16:**
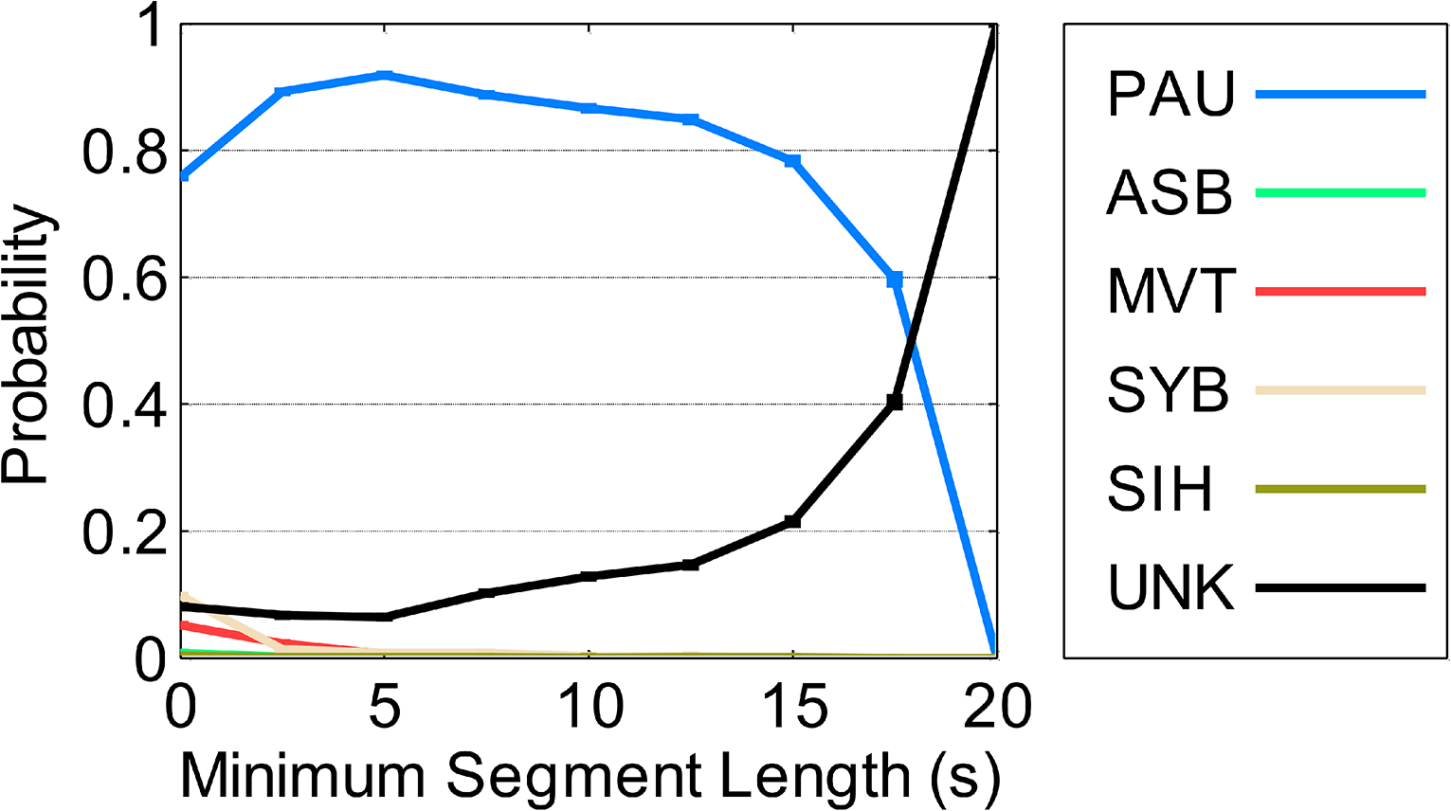
Confusion of SC1 on samples with consensus pattern of pause as a function of segment length. SYB = synchronous-breathing, ASB = asynchronous-breathing, SIH = sigh, PAU = pause, MVT = movement artifact, UNK = unknown. A probability of 1 for PAU indicates no confusion. Lower PAU probabilities indicate increased confusion. Standard deviations of all probabilities were < 0.01.

Note that segment length had no effect on the confusion matrix for SC2 and SC3, but for SC1, confusion of PAU varied with segment length. Fig 16 illustrates that SC1 confused PAU segments longer than 15 s with UNK, and this confusion increased with segment length.

## Discussion

This paper describes a novel set of tools for the manual analysis of infant respiratory inductive plethysmography (RIP) data. The tool set includes 5 components:

- A set of clear, concise definitions of RIP patterns, and scoring rules based on uncalibrated RIP data. These definitions and rules make it possible to fully characterize an infant’s respiratory behavior across extended periods of time, thus enabling the analysis of long data records required for the study of Postoperative Apnea (POA).
- An interactive, computer application (RIPScore) that supports the application of the scoring rules to infant data in an efficient manner. RIPScore incorporates the capability to track the rate at which scorers analyze data; providing the objective measurement of the time required to analyze a dataset.
- A library of “true-pattern” segments representing each of the 6 RIP patterns, used for training, assessment of scorer performance, and development of evaluation methods.
- A formal training protocol based on the interactive, completely automated RIPScore Training and Evaluation Modes. This protocol allows scorers from varied backgrounds to become proficient with RIPScore and the scoring protocol, and reach a standardized performance level similar to that of an expert. This training protocol obviates the requirement of certified sleep laboratory technicians, helping to reduce analysis costs, while increasing the feasibility of recruiting new scorers.
- A method to monitor the ongoing performance of scorers over time. This quality control measure allows the monitoring of scorers throughout the study to ensure they maintain a standardized performance. An advantage of this method is the early identification of underperforming scorers, which might allow for corrective action to assure the analysis quality.

The validation experiment demonstrates that analysis with these tools is accurate, efficient, and has high intra- and inter- scorer repeatability. These characteristics make our tools appropriate for studying respiratory conditions where large datasets (e.g., POA), and multiple scorers (e.g., longitudinal, multicenter trials) are a necessity.

### Comparison to Existing Manual Scoring Tools

Commercially available scoring software is designed to analyze data based on the AASM scoring rules [10]. Using this software, scorers analyze data records and detect clinically relevant respiratory events such as central, obstructive, and mixed apnea. This analysis does not provide a comprehensive description of respiratory behavior as a function of time, because it focuses only on detecting and scoring isolated segments of data. As a result, the AASM analysis ignores potentially informative data segments. For example short respiratory pauses are not considered, even though they are more frequent in infants with POA than in controls [20]. Additionally, the AASM rules require scorers to scroll throughout long records and visually detect candidate events. This strategy is prone to fatigue, leading to missed detections and increased variability.

In contrast, analysis with RIPScore requires that signals are analyzed continuously, on a sample-by-sample basis. An advantage of this continuous analysis is that the complete data record is classified. As a result, the instantaneous respiratory pattern is fully characterized as a function of time, enabling a comprehensive signals and systems analysis approach to the study of disorders of respiration such as POA. Additionally, the focus of scorers is changed from visual detection of events to classification of data segments. This design requires scorers to analyze all data segments and so it is not possible to miss events. Moreover, contrary to the AASM rules, our tools impose no arbitrary segment length definitions that may exclude short but relevant segments [20].

### Training of Scorers

RIPScore provides an interactive Training Mode that familiarizes trainees with the interface, provides practice in scoring with immediate feedback using simulated data, and evaluates their performance. Three scorers with very varied backgrounds were trained in this way. All trainees reached the desired performance after four 2-hour training/evaluation sessions. Thus, by the end of training, all 3 scorers regardless of their clinical expertise, reached a standardized performance similar to that of the experienced reference scorer (REF). This implies that for large projects requiring multiple scorers, it should be possible to efficiently train a cadre of naive scorers to have performance similar to that of an expert.

### Accuracy and Consistency

The scorers used our tools to carry out a comprehensive manual analysis of the pre-processed validation dataset, comprising 21 infant data records that incorporated quality control segments with known “true-patterns”; a total of 125 hours of data were manually analyzed twice per scorer. The ongoing accuracy and consistency of each scorer was assessed by analyzing the RIP patterns assigned to the quality control “true-pattern” segments. All scorers maintained a high, relatively constant overall accuracy throughout the analysis of the 42 data records. The consistency of the two scorers with clinical expertise (SC1 and SC2) was nearly constant throughout, while the consistency of the third, non-clinical scorer (SC3) quickly rose to a level similar to that of the other two scorers after 10 data records. The high, nearly constant values of overall accuracy and consistency are evidence that the training protocol was effective, since scorers were able to achieve and maintain the desired performance level throughout.

It is noteworthy that for the PAU pattern, SC3 had lower accuracy and consistency for most of the data records, suggesting that a minimum clinical expertise with infant respiratory patterns may be necessary to maintain the desired performance. Fig 14A and 14B suggest that even though the PAU-specific performance of SC3 was lower than expected, the initial 3 values of accuracy and consistency were likely influenced by training since they matched the values of SC1 and SC2. It was until after the third record that the performance of SC3 dropped. It is possible that an intervention at this point might have mitigated deterioration in PAU-specific performance.

### Scoring Rate

We measured the rate at which scorers analyzed infant data throughout the study. Scoring was efficient, occurring at a rate of at least 1 hr of data analyzed in 1 hr. Scorers with no previous scoring experience gradually increased their rate, with no loss of either accuracy or consistency. In contrast, the sleep laboratory technician (SC2) maintained a constant rate. We believe that the design of the RIPScore Scoring Mode interface, which only required a single cursor selection and one key stroke to score a segment, facilitated this efficient analysis rate.

### Repeatability of the Manual Analysis

The repeatability analysis showed that the two scorers with clinical background had very good intra-scorer repeatability, similar to that of REF. The scorer with no clinical expertise had a slightly lower intra-repeatability but it was still substantial.

The inter-scorer repeatability was very good in most categories. Indeed, the overall inter-scorer repeatability was much higher (*κ* = 0.65) than that reported between expert scorers from sleep laboratories using conventional scoring tools (*κ* = 0.31) [22]. For the particular pattern of PAU, intra- (0.74 ≤ *κ* ≤ 0.79) and inter-scorer (*κ* = 0.65) repeatability were substantial, which is relevant for the study of apnea. UNK was the pattern with lowest repeatability. Intra- and inter-scorer repeatability were also low for SIH, the only pattern requiring a breath-by-breath manual analysis.

### Confusion of Patterns

Analysis of the confusion among RIP patterns found that SYB, ASB, SIH, and PAU were not often confused with other patterns. MVT and UNK were frequently confused with each other. This was the main reason for the low repeatability of UNK. This was expected since UNK grouped ambiguous patterns and segments of low signal quality. Even though this was a misclassification, both MVT and UNK correspond to corrupted data segments meant to be excluded from further analyses.

Additionally, we evaluated the influence of segment length on confusion, and found that segment length was a factor for only one scorer (SC1), who confused PAU segments longer than 15 s with UNK. A possible explanation is that SC1 might have interpreted long periods without respiratory movements as missing data resulting from technical problems, rather than as long PAU segments.

### Implementation and Availability

RIPScore was implemented in MATLAB (The MathWorks Inc., Natick, MA, USA), compiled as a standalone application, and installed on the scorers’ personal computers for the validation study. RIPScore and the pre-processing algorithm have been made available as open source, free of charge software; the manual (S1 Document) and complete function repository (S1 Source Code) are in GitHub (www.github.com/McCRIBS). The standalone application is available from the authors upon request.

### Future Work

A difference between the manual scoring tools presented in this work and the AASM methodology is that respiratory behavior is classified in terms of 6 mutually exclusive patterns, instead of the occurrence respiratory events such as apnea. At present, no direct link has been established between the 6 patterns and respiratory events. However, the patterns could be post-processed to identify respiratory events. For instance, a PAU with duration longer than a threshold (e.g., 15 s) would define a central apnea. Similarly, a combination of PAU with ASB would define a mixed obstructive apnea. Future work is necessary to evaluate the utility of a secondary set of rules based on pattern post-processing for the identification of clinically relevant respiratory events.

A direct application of the tools presented in this paper is the study of POA, and its relation to postoperative respiratory patterns. There is a variety of evidence suggesting that infants who experience POA have abnormal postoperative respiratory patterns [2, 20, 48]. Based on this, one could hypothesize that postoperative respiratory patterns may have information that is predictive of POA. The manual scoring tools from this paper could be used to investigate this hypothesis because they provide the means needed to comprehensively describe the respiratory patterns. Thus, for example, it would be straightforward to extract features from the manual scoring results related to information of the respiratory patterns such as the frequency of pauses, the proportion of time spent in each pattern, the relative proportion of synchronous- versus asynchronous-breathing, or the temporal sequence of patterns. Future work will investigate these and other features extracted from the respiratory patterns, and their ability to predict POA.

### Significance

The tools for manual scoring introduced in this paper provide a comprehensive framework for the analysis of infant RIP data. These tools offer a significant advance in the study of respiratory behavior by providing: a comprehensive analysis method for large data sets, a means for the training and standardization of scorers, a method for the ongoing monitoring of scorer consistency and accuracy, and open source access to software and data sets.

#### Comprehensive Analysis

The tools provide a clear, concise definition of RIP patterns, and a software application (RIPScore) to locate these patterns along data records. The analyzed data record represents a sample-by-sample characterization of respiratory behavior as a continuous time series of patterns. All data points are classified and thereby significant segments are not missed. This approach facilitates the study of respiratory behavior from a signals and systems perspective by enabling the study of the temporal correlation between POA and the varied respiratory patterns (e.g., the relation between pause frequency and POA). The development of models that predict POA occurrence becomes possible, and preemptive interventions to enable preventive actions may follow.

#### Training & Standardization

The tools can be used to train any person to be a scorer, regardless of background, to achieve a standardized performance level similar to that of an expert. The ability to quickly train new scorers recruited from varied backgrounds increases the availability of potential scorers, thus helping to reduce the analysis cost by obviating the need for certified sleep laboratory technicians.

#### Monitoring of Scorer Performance

Another major contribution of this work is that the manual scoring tools make possible multicenter and longitudinal studies requiring multiple scorers. Conventional scoring tools have heretofore limited these types of study because of a low intra- and inter- scorer repeatability [22]. Intra-scorer repeatability is important to ensure that scorers maintain consistency throughout the period of data analysis. Inter-scorer repeatability is necessary to maintain the consistency of results among multiple scorers. The quality control method introduced in this work evaluates the ongoing scorer performance on a record-by-record basis. This quality control tool can identify underperforming scorers at any time throughout the duration of the study. This timely identification enables investigators to take corrective actions (e.g., additional training, scorer replacement) to maintain the desired performance. This ability will in turn help to reduce intra- and inter-scorer variability.

#### Open Source Access

Importantly, all the tools presented in this work are openly available to researchers interested in the analysis of respiratory patterns using RIP, and the study of POA. In addition to the RIP pattern definitions, scoring rules, representative examples, and training protocol described in this manuscript; the software, including RIPScore and the pre-processing method for quality control, are freely available (www.github.com/McCRIBS). Finally, the library of “true-pattern” data segments, the complete dataset from infants at risk of POA, the training sessions, and analysis results from the 4 scorers are available from the Dryad Digital Repository (doi:10.5061/dryad.72dk5).

## Conclusion

The tools presented in this work provide an excellent framework for study of infant respiratory behavior because they: (i) classify all respiratory patterns as a time series, (ii) standardize scorer performance using a training protocol which employs simulated data, (iii) monitor scoring repeatability by providing an ongoing quality control supervision of scorers, and (iv) are openly available and can be readily used in any study involving RIP.

## Supporting Information

S1 Fig Evaluation of manual scoring of Movement Artifact. (A) Accuracy (Fleiss’ *κ*); (B) consistency (Fleiss’ *κ*); and (C) rate (hours of data per hour of scoring). Results are shown for the 42 data records analyzed (21 files scored twice).
(TIF)

S2 Fig Evaluation of manual scoring of Unknown. (A) Accuracy (Fleiss’ *κ*); (B) consistency (Fleiss’ *κ*); and (C) rate (hours of data per hour of scoring). Results are shown for the 42 data records analyzed (21 files scored twice).
(TIF)

S3 Fig Evaluation of manual scoring of Synchronous-Breathing. (A) Accuracy (Fleiss’ *κ*); (B) consistency (Fleiss’ *κ*); and (C) rate (hours of data per hour of scoring). Results are shown for the 42 data records analyzed (21 files scored twice).
(TIF)

S4 Fig Evaluation of manual scoring of Asynchronous-Breathing. (A) Accuracy (Fleiss’ *κ*); (B) consistency (Fleiss’ *κ*); and (C) rate (hours of data per hour of scoring). Results are shown for the 42 data records analyzed (21 files scored twice).
(TIF)

S5 Fig Evaluation of manual scoring of Sigh. (A) Accuracy (Fleiss’ *κ*); (B) consistency (Fleiss’ *κ*); and (C) rate (hours of data per hour of scoring). Results are shown for the 42 data records analyzed (21 files scored twice).
(TIF)

S6 Fig Individual confusion matrix of scorer SC1. Conditional probability of each respiratory inductive plethysmography (RIP) pattern for samples with the consensus pattern of: (A) synchronous-breathing (SYB), (B) asynchronous-breathing (ASB), (C) pause (PAU), (D) sigh (SIH), (E) movement artifact (MVT), and (F) unknown (UNK). When there is no confusion, the consensus pattern has a probability of 1 and the others have probabilities of 0. During total confusion all patterns have equal probabilities. Standard deviations of all probabilities were < 0.01.
(TIF)

S7 Fig Individual confusion matrix of scorer SC2. Conditional probability of each respiratory inductive plethysmography (RIP) pattern for samples with the consensus pattern of: (A) synchronous-breathing (SYB), (B) asynchronous-breathing (ASB), (C) pause (PAU), (D) sigh (SIH), (E) movement artifact (MVT), and (F) unknown (UNK). When there is no confusion, the consensus pattern has a probability of 1 and the others have probabilities of 0. During total confusion all patterns have equal probabilities. Standard deviations of all probabilities were < 0.01.
(TIF)

S8 Fig Individual confusion matrix of scorer SC3. Conditional probability of each respiratory inductive plethysmography (RIP) pattern for samples with the consensus pattern of: (A) synchronous-breathing (SYB), (B) asynchronous-breathing (ASB), (C) pause (PAU), (D) sigh (SIH), (E) movement artifact (MVT), and (F) unknown (UNK). When there is no confusion, the consensus pattern has a probability of 1 and the others have probabilities of 0. During total confusion all patterns have equal probabilities. Standard deviations of all probabilities were < 0.01.
(TIF)

S1 Document RIPScore user manual. A guide on how to install, run, and configure RIPScore. The manual also describes the format of files read and produced by RIPScore.
(PDF)

S1 Source Code RIPScore source code and test data. McGill CardioRespiratory Infant Behavior Software (McCRIBS) source code including RIPScore and ancillary functions. The package also includes two test data records.
(ZIP)
