# Correction: Scoring Tools for the Analysis of Infant Respiratory Inductive Plethysmography Signals

**DOI:** 10.1371/journal.pone.0156477

**Published:** 2016-05-25

**Authors:** The PLOS ONE Staff

There is an error in the caption for Fig 1, “Elements of the RIP Score interface.” Please see the correct Fig 1 caption here.

**Fig 1:**
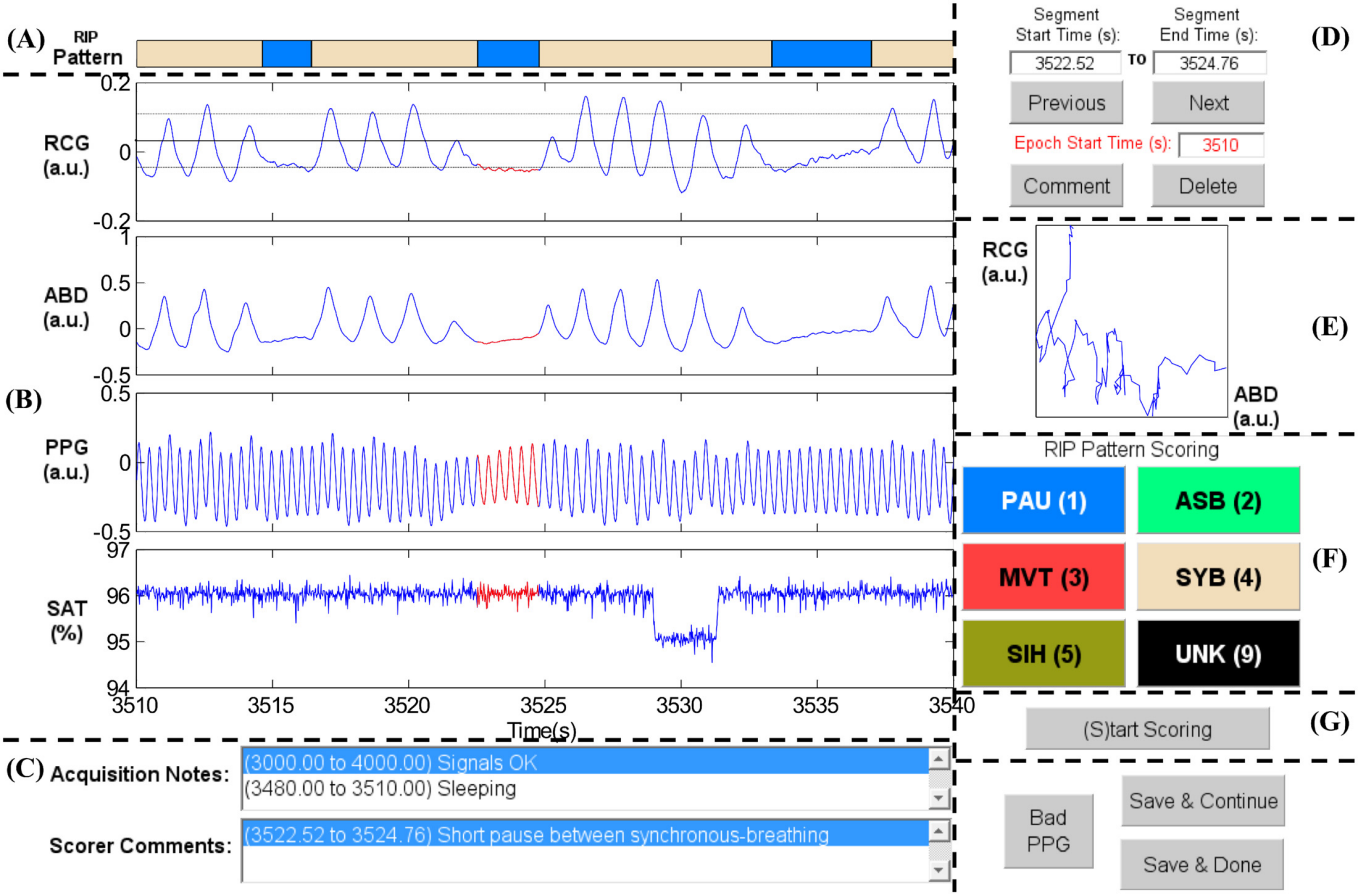
Elements of the RIPScore interface. (A) Respiratory Inductive Plethysmography (RIP) Pattern; (B) Signals from ribcage (RCG), abdomen (ABD), photoplethysmograph (PPG), and blood oxygen saturation (SAT); (C) Notes; (D) Segment and Epoch Control; (E) Lissajous Figure; (F) RIP Pattern Scoring; and (G) Mode Control. The epoch shows a representative example of Pause (PAU). The quasi-sinusoidal pattern in RCG and ABD stops during the PAU highlighted in red. The horizontal dotted cursors in RCG show an estimated variation of ± 90% of the amplitude of the breath preceding the PAU. Note that these cursors do not take into account low frequency trends, and so are only an approximate reference. a.u. = arbitrary units.

There is also an error in the “RIP Score” subsection of the Tools for Manual Scoring section. The following text was omitted from the “Main Screen” heading in the “RIP Score” subsection and incorrectly included in the caption for Fig 1. The publisher apologizes for the error.

*(A) RIP Pattern*: a color-coded bar showing the RIP pattern assigned by the scorer at each time; *(B) Signals*: plots of the cardiorespiratory signals including ribcage (RCG), abdomen (ABD), photoplethysmograph (PPG), and blood oxygen saturation (SAT). Clicking on a breath from RCG or ABD plots three horizontal cursors, one at the estimated breath’s amplitude, and two at ± 90% of that amplitude. Note that these cursors are not an exact amplitude reference for the epoch because they do not take into account low frequency trends frequently observed in RIP signals [35]; *(C)Notes*: text boxes showing time stamped notes made during data acquisition, and comments entered by the scorer during analysis; *(D) Segment and Epoch Control*: text boxes showing the start and end times for the current segment (highlighted in red in *Signals*); command buttons to add a “Comment” or “Delete” the RIP pattern assigned to the current segment; command buttons to scroll through epochs (“Previous”, “Next”), and a text box with the start time of the current epoch; *(E) Lissajous Figure*: a plot of RCG versus ABD for the current segment to aid the user in evaluating thoraco-abdominal synchrony. During breathing, the plot will be an ellipse tilted to the right for a phase less than 90 degrees, a circle for a phase of 90 degrees, and an ellipse tilted to the left for a phase greater than 90 degrees; *(F) RIP Pattern Scoring*: color-coded command buttons that assign a RIP pattern to the current segment; each button may also be activated by hitting the corresponding keyboard “hot-key” defined by the character in parenthesis for each button (e.g., the hot-key for Pause is ‘1’); *(G) Mode Control*: command button to switch between scoring and visualization mode.
