# Supplementary figures and images for: Scoring Tools for the Analysis of Infant Respiratory Inductive Plethysmography Signals
Source: PLoS One. 2015 Jul 28;10(7):e0134182. doi: 10.1371/journal.pone.0134182 (PMC4517879; doi:10.1371/journal.pone.0134182)

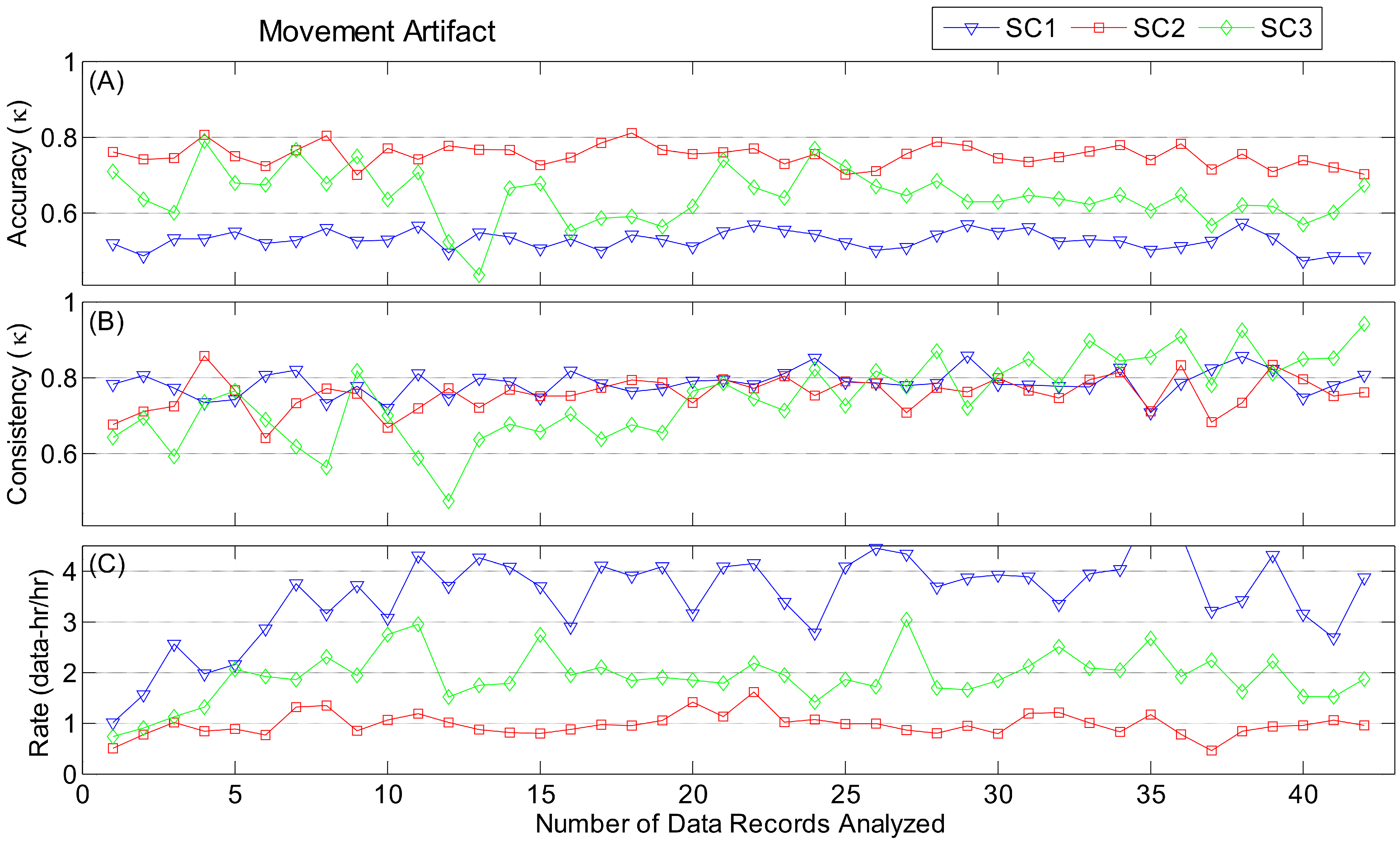

Supplement: S1 Fig — (A) Accuracy (Fleiss’ κ); (B) consistency (Fleiss’ κ); and (C) rate (hours of data per hour of scoring). Results are shown for the 42 data records analyzed (21 files scored twice). (TIF) [file pone.0134182.s001.tif]

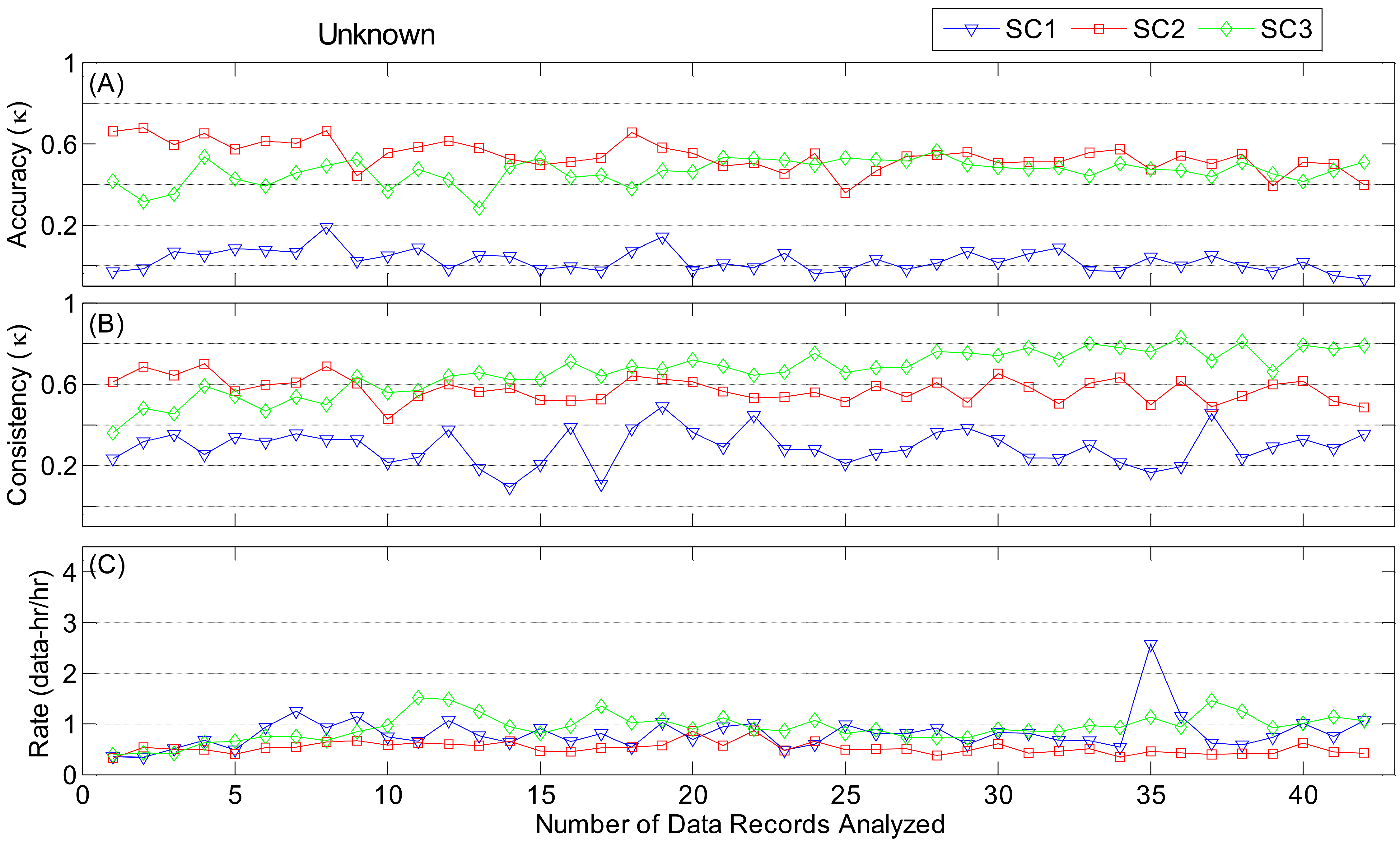

Supplement: S2 Fig — (A) Accuracy (Fleiss’ κ); (B) consistency (Fleiss’ κ); and (C) rate (hours of data per hour of scoring). Results are shown for the 42 data records analyzed (21 files scored twice). (TIF) [file pone.0134182.s002.tif]

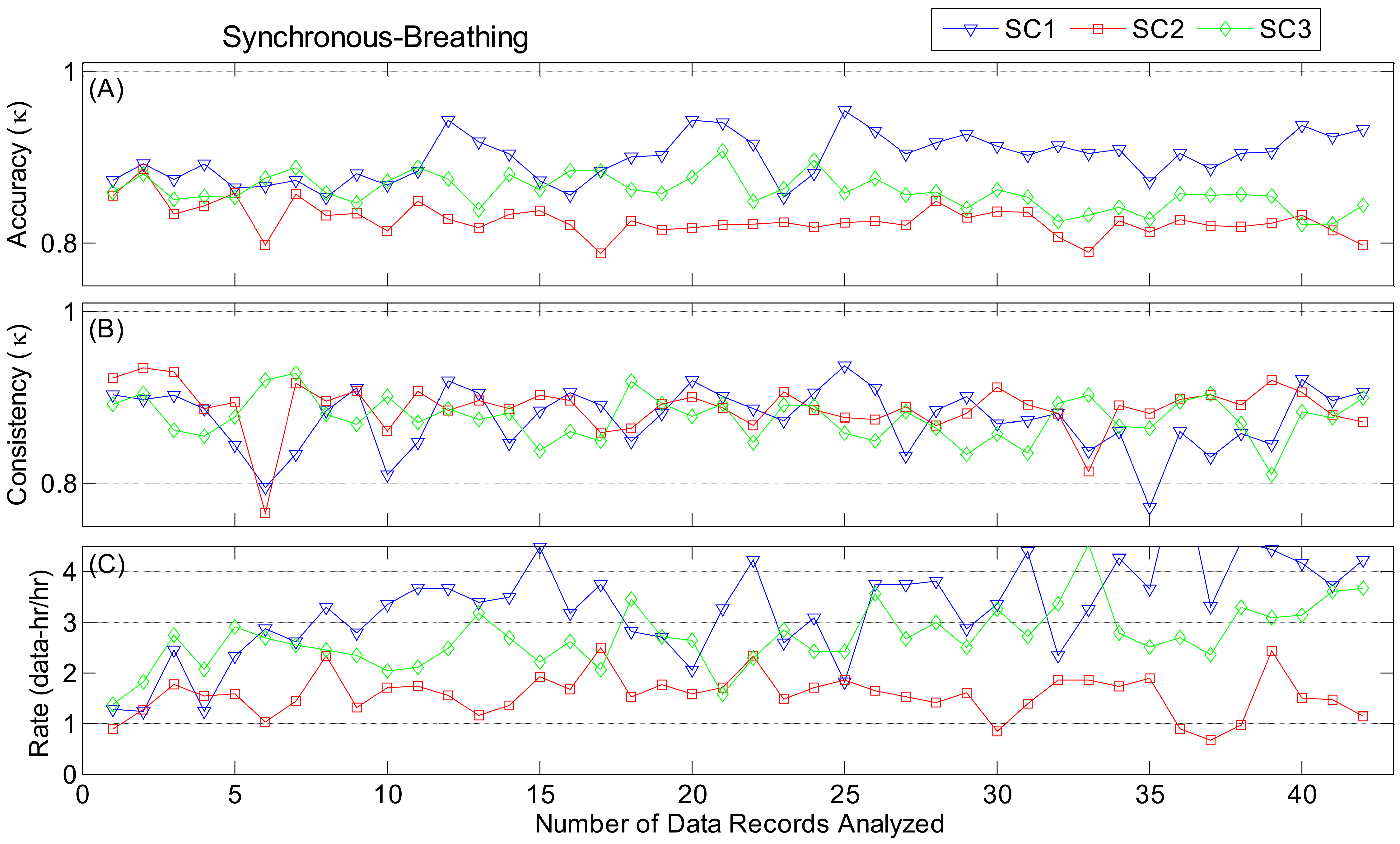

Supplement: S3 Fig — (A) Accuracy (Fleiss’ κ); (B) consistency (Fleiss’ κ); and (C) rate (hours of data per hour of scoring). Results are shown for the 42 data records analyzed (21 files scored twice). (TIF) [file pone.0134182.s003.tif]

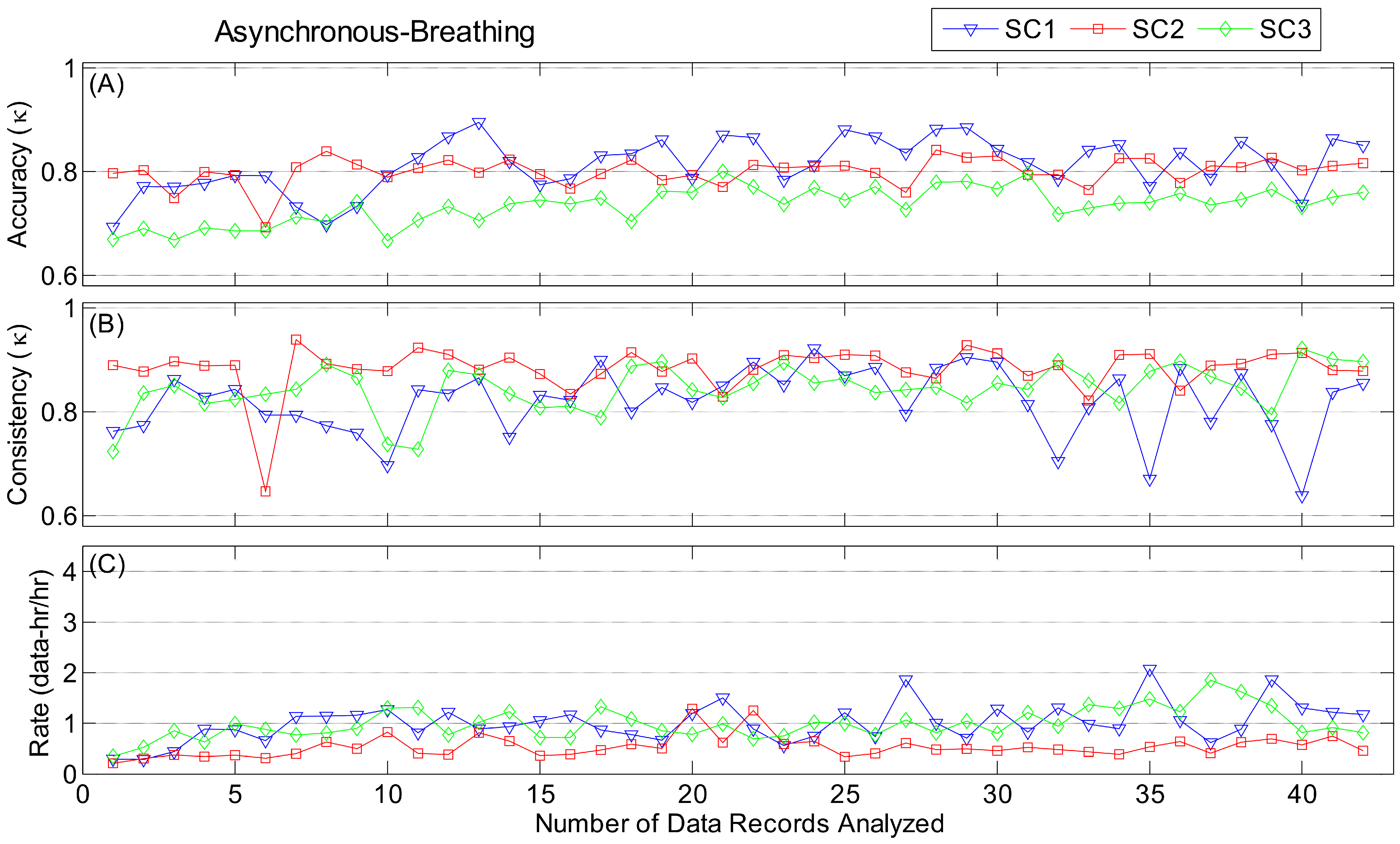

Supplement: S4 Fig — (A) Accuracy (Fleiss’ κ); (B) consistency (Fleiss’ κ); and (C) rate (hours of data per hour of scoring). Results are shown for the 42 data records analyzed (21 files scored twice). (TIF) [file pone.0134182.s004.tif]

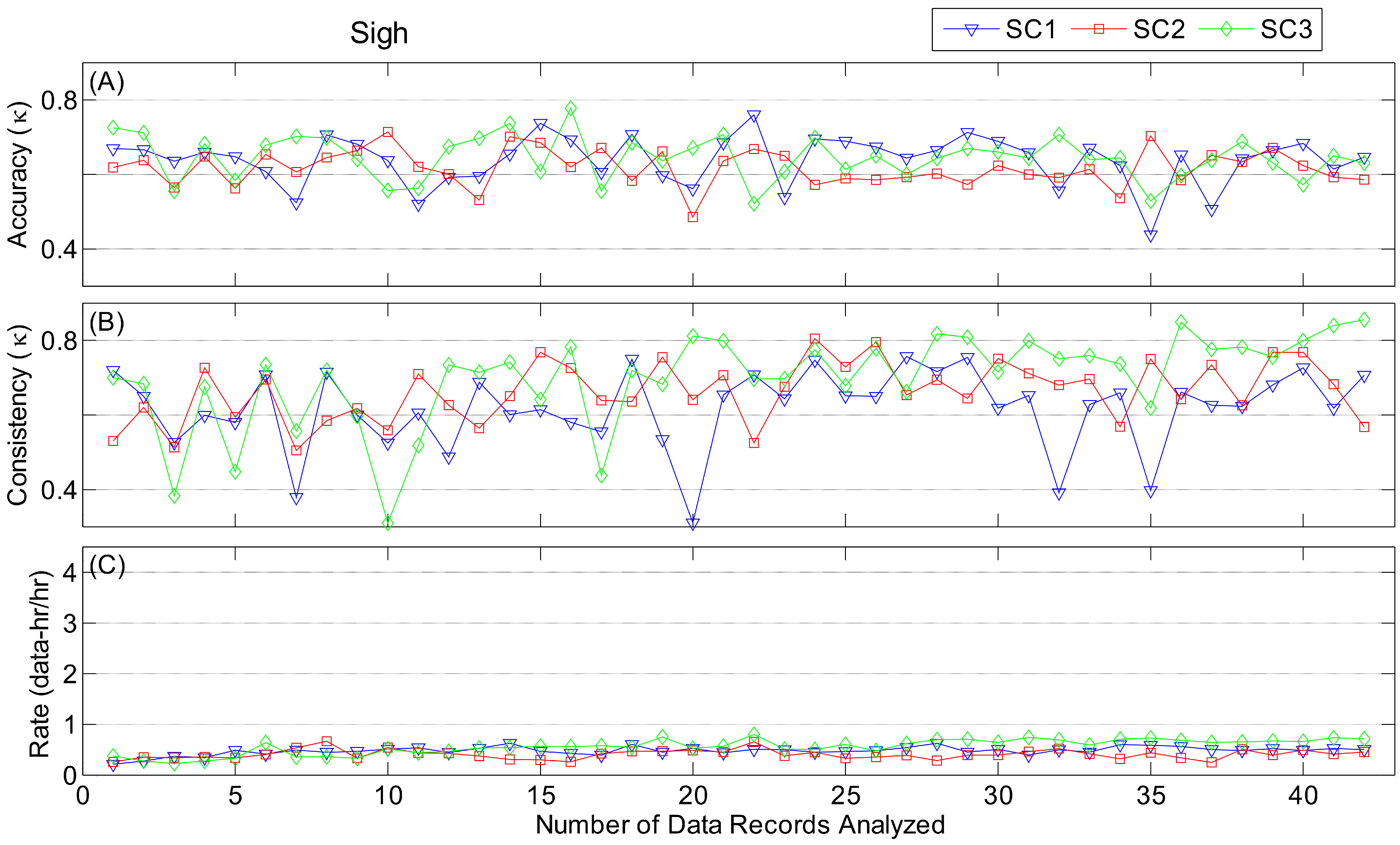

Supplement: S5 Fig — (A) Accuracy (Fleiss’ κ); (B) consistency (Fleiss’ κ); and (C) rate (hours of data per hour of scoring). Results are shown for the 42 data records analyzed (21 files scored twice). (TIF) [file pone.0134182.s005.tif]

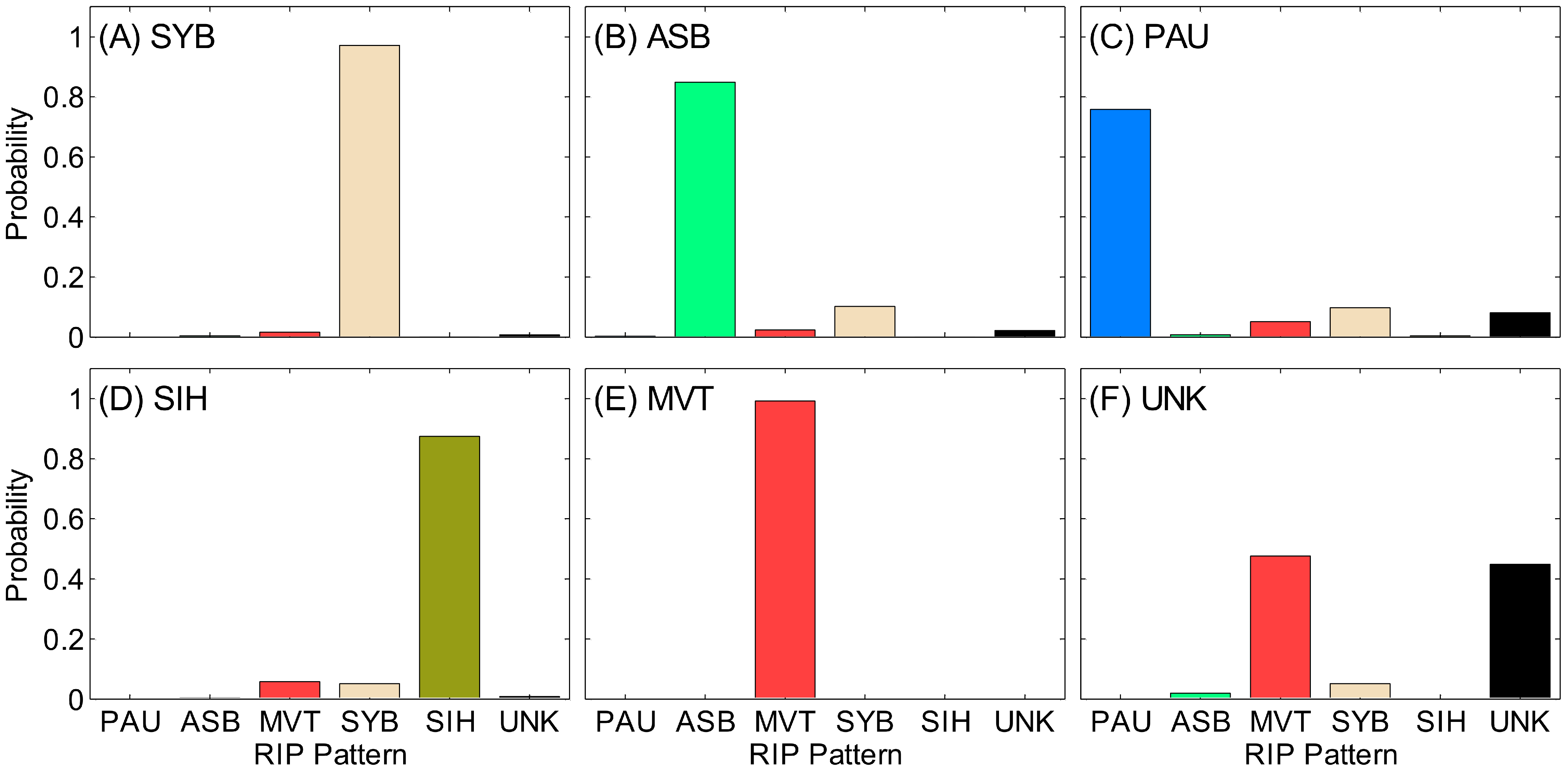

Supplement: S6 Fig — Conditional probability of each respiratory inductive plethysmography (RIP) pattern for samples with the consensus pattern of: (A) synchronous-breathing (SYB), (B) asynchronous-breathing (ASB), (C) pause (PAU), (D) sigh (SIH), (E) movement artifact (MVT), and (F) unknown (UNK). When there is no confusion, the consensus pattern has a probability of 1 and the others have probabilities of 0. During total confusion all patterns have equal probabilities. Standard deviations of all probabilities were < 0.01. (TIF) [file pone.0134182.s006.tif]

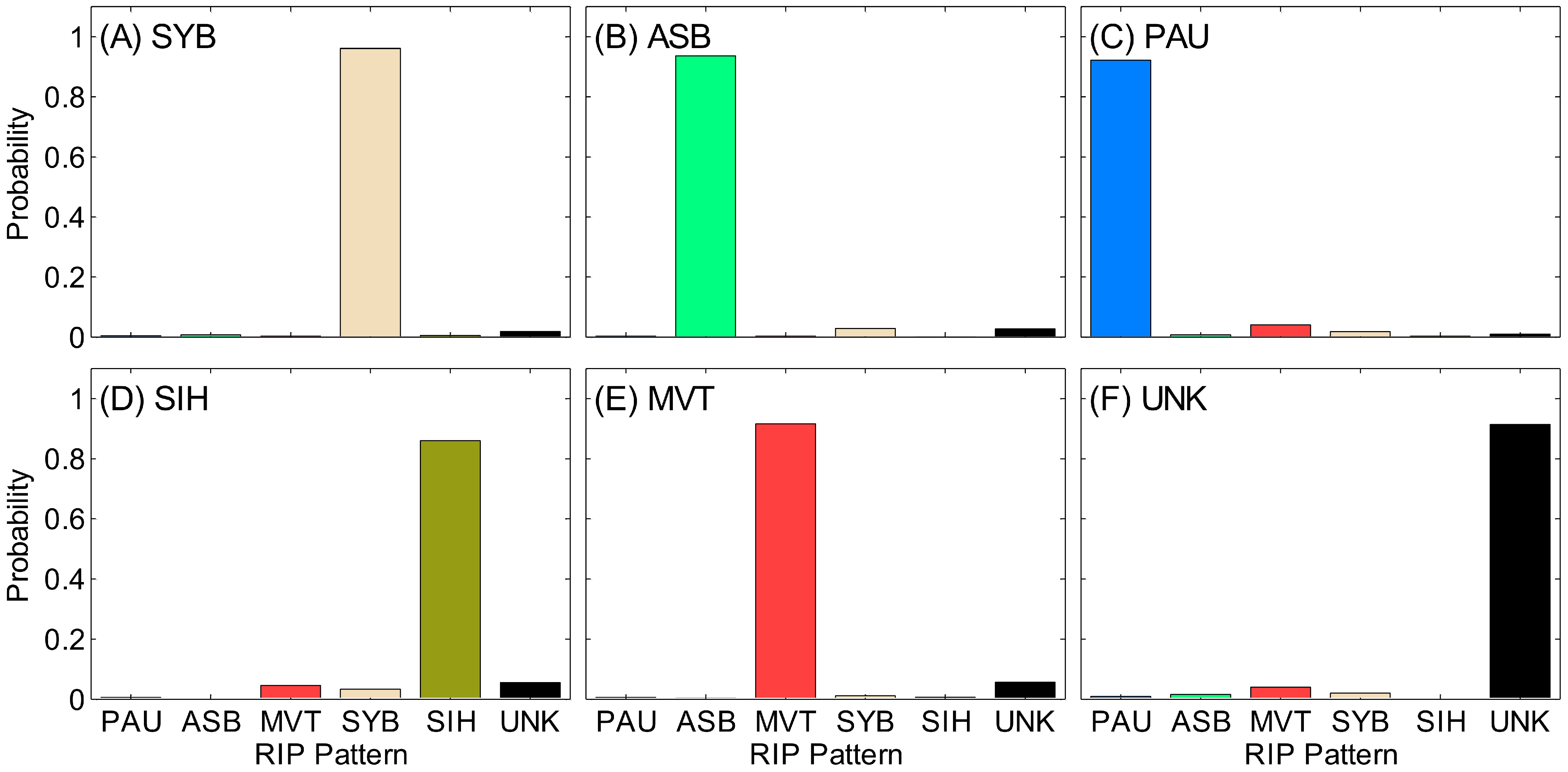

Supplement: S7 Fig — Conditional probability of each respiratory inductive plethysmography (RIP) pattern for samples with the consensus pattern of: (A) synchronous-breathing (SYB), (B) asynchronous-breathing (ASB), (C) pause (PAU), (D) sigh (SIH), (E) movement artifact (MVT), and (F) unknown (UNK). When there is no confusion, the consensus pattern has a probability of 1 and the others have probabilities of 0. During total confusion all patterns have equal probabilities. Standard deviations of all probabilities were < 0.01. (TIF) [file pone.0134182.s007.tif]

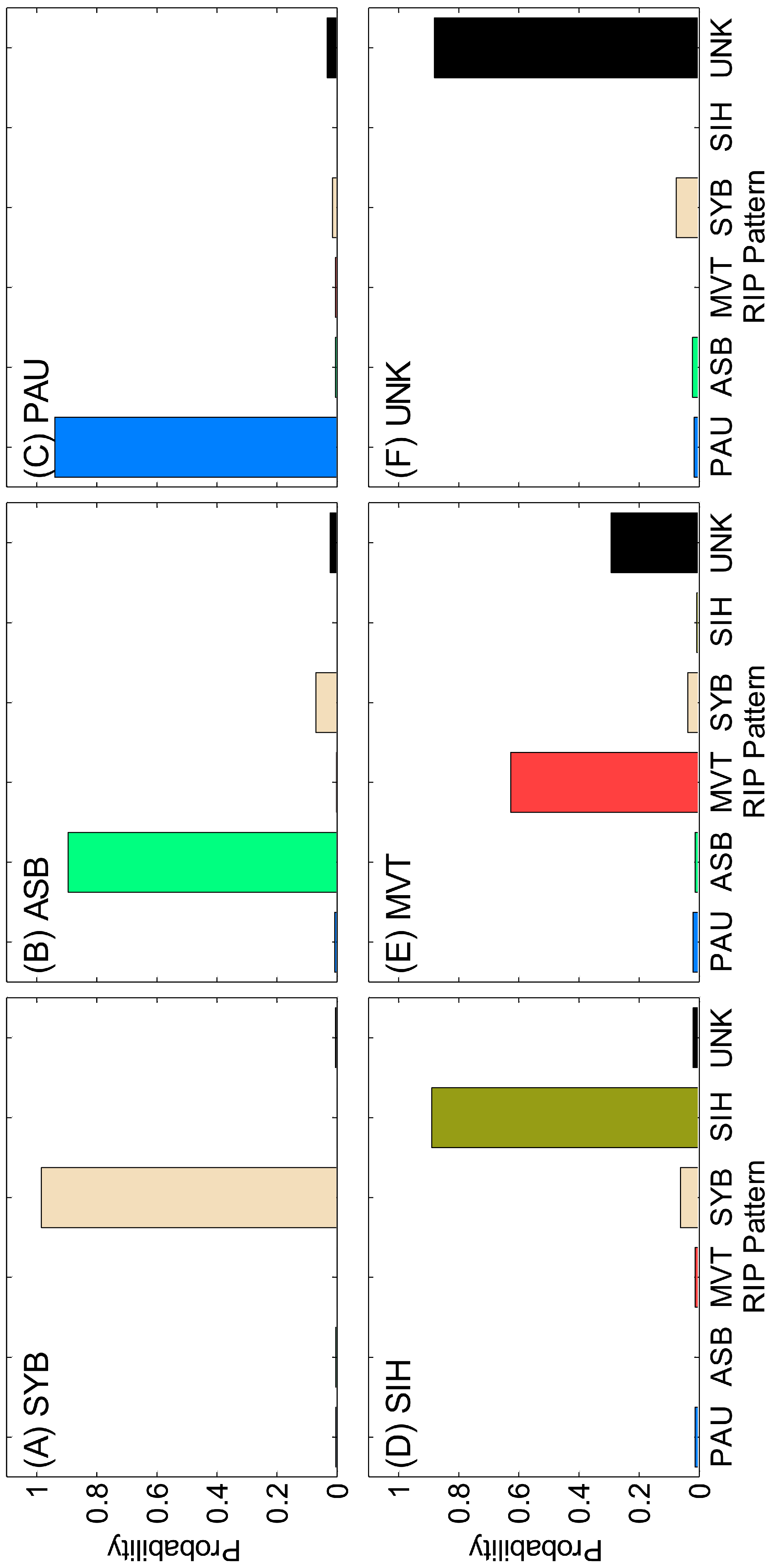

Supplement: S8 Fig — Conditional probability of each respiratory inductive plethysmography (RIP) pattern for samples with the consensus pattern of: (A) synchronous-breathing (SYB), (B) asynchronous-breathing (ASB), (C) pause (PAU), (D) sigh (SIH), (E) movement artifact (MVT), and (F) unknown (UNK). When there is no confusion, the consensus pattern has a probability of 1 and the others have probabilities of 0. During total confusion all patterns have equal probabilities. Standard deviations of all probabilities were < 0.01. (TIF) [file pone.0134182.s008.tif]
