# Supplementary material for: Scoring Tools for the Analysis of Infant Respiratory Inductive Plethysmography Signals
Source: PLoS One. 2015 Jul 28;10(7):e0134182. doi: 10.1371/journal.pone.0134182 (PMC4517879; doi:10.1371/journal.pone.0134182)
Supplement: S1 Document — A guide on how to install, run, and configure RIPScore. The manual also describes the format of files read and produced by RIPScore. (PDF) [file pone.0134182.s009.pdf]

RIPScore

(v1.2)

User Manual

Copyright © 2015, Carlos Alejandro Robles Rubio, Karen A. Brown, and Robert E. Kearney
McGill University

Table of Contents

I. LICENSE 3

II. ACKNOWLEDGEMENT 4

III. QUICK START 5

A. Description 5
B. Required Libraries 5
C. Run RIPScore 5

IV. CONFIGURATION 8

V. DATA FILES 12

A. Input 12
B. Output 12

VI. LIST OF ACRONYMS 16

VII. REFERENCES 17

I. License

Copyright (c) 2015, Carlos Alejandro Robles Rubio, Karen A. Brown, and Robert E. Kearney,
McGill University
All rights reserved.

Redistribution and use in source and binary forms, with or without modification, are permitted provided that the following conditions are met:

1. Redistributions of source code must retain the above copyright notice, this list of conditions and the following disclaimer.

2. Redistributions in binary form must reproduce the above copyright notice, this list of conditions and the following disclaimer in the documentation and/or other materials provided with the distribution.

THIS SOFTWARE IS PROVIDED BY THE COPYRIGHT HOLDERS AND CONTRIBUTORS "AS IS" AND ANY EXPRESS OR IMPLIED WARRANTIES, INCLUDING, BUT NOT LIMITED TO, THE IMPLIED WARRANTIES OF MERCHANTABILITY AND FITNESS FOR A PARTICULAR PURPOSE ARE DISCLAIMED. IN NO EVENT SHALL THE COPYRIGHT HOLDER OR CONTRIBUTORS BE LIABLE FOR ANY DIRECT, INDIRECT, INCIDENTAL, SPECIAL, EXEMPLARY, OR CONSEQUENTIAL DAMAGES (INCLUDING, BUT NOT LIMITED TO, PROCUREMENT OF SUBSTITUTE GOODS OR SERVICES; LOSS OF USE, DATA, OR PROFITS; OR BUSINESS INTERRUPTION) HOWEVER CAUSED AND ON ANY THEORY OF LIABILITY, WHETHER IN CONTRACT, STRICT LIABILITY, OR TORT (INCLUDING NEGLIGENCE OR OTHERWISE) ARISING IN ANY WAY OUT OF THE USE OF THIS SOFTWARE, EVEN IF ADVISED OF THE POSSIBILITY OF SUCH DAMAGE.

II. Acknowledgement

The development of RIPScore was supported in part by the Natural Sciences and Engineering Research Council of Canada. The work of Carlos Alejandro Robles Rubio was supported in part by the Mexican National Council for Science and Technology, and in part by the Queen Elizabeth Hospital of Montreal Foundation Chair in Pediatric Anesthesia. Karen A. Brown was supported in part by the Queen Elizabeth Hospital of Montreal Foundation Chair in Pediatric Anesthesia.

III.Quick Start

A. Description

RIPScore is an open source, interactive software application for manual scoring of infant Respiratory Inductive Plethysmography (RIP) data. A detailed description of the analysis that can be performed using RIPScore is in [1]. RIPScore was developed in MATLAB™ (The MathWorks, Inc., Natick, MA, USA).

B. Required Libraries

It is necessary to obtain the following libraries to use RIPScore.

1) McCRIBS (McGill CardioRespiratory Infant Behavior Software) tools. These tools are open source functions developed in MATLAB™, and are available for download free of charge at github.com/McCRIBS. Download the tools and save them in the directory `C:\McCRIB\McCRIBS`.
2) A library of "true-pattern" segments as defined in [1]. This library is available for download free of charge at datadryad.org (for details see [1]). Download the data and save them in the directory `C:\McCRIB\Data`.

C. Run RIPScore

The following steps provide a guide to start using RIPScore with default configuration parameters.

1) Open MATLAB™.
2) Setup the McCRIBS working environment in MATLAB™. by running this code:

```
%Path to the McCRIBS tools directory (downloaded from github.com)
SourceCodeRoot='C:\McCRIB\McCRIBS';
%Path to the Data directory (downloaded from datadryad.org)
DataRoot='C:\McCRIB\Data';

%Setup the working environment
cd([SourceCodeRoot '\Utilities']);
setMcCRIBS_Env(SourceCodeRoot,DataRoot)
McCRIB_DATA_ROOT=getenv('McCRIB_DATA_ROOT'); %The Data directory.
%Go to RIPScore root directory
cd([SourceCodeRoot '\RIPSCORE\RIPScore']);
```

3) Start the application by typing `RIPScore` in the MATLAB command window.
4) RIPScore will load with the pre-defined, default configuration (Blind Scorer Mode). Click `OK` in the following screen:

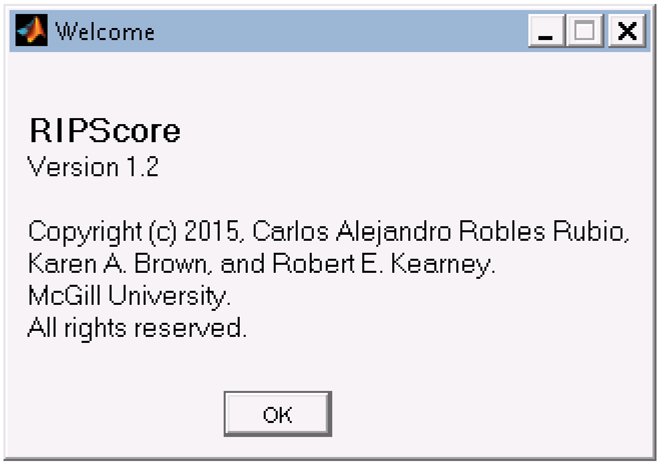

5) RIPScore will then ask for Scorer Identification. Select `Add New…`:

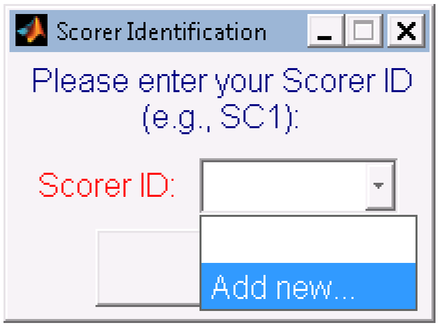

6) Type the Scorer ID in the text box (e.g., `TEST`), and click `OK`:

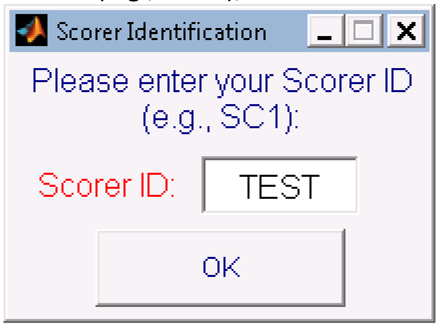

7) RIPScore will display the number of files available for scorer `TEST` to analyze. Click `OK`.

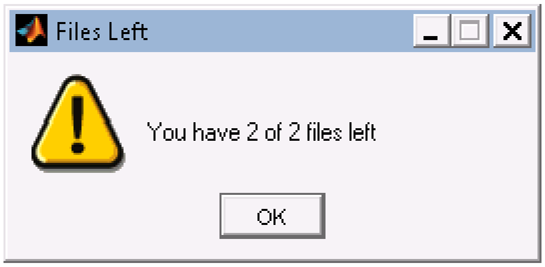

8) At this point RIPScore will randomly select one of the files left and load it for scorer `TEST` to analyze.
9) Since this is the first time scorer `TEST` will analyze the current file, RIPScore will output the following message:

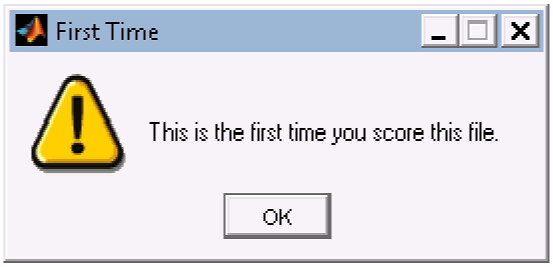

10) Click `OK`. RIPScore will load in Visualization Mode. Scorer `TEST` is ready to analyze the data.
11) For a detailed description of the main screen controls, and their functionalities see [1].
12) In this default configuration, the scoring results are stored at:
`C:\McCRIB\McCRIBS\RIPSCORE\scored`

13) Also, the sample data records are loaded from:
`C:\McCRIB\McCRIBS\RIPSCORE\data`

These records constitute brief segments of infant cardiorespiratory data, pre-processed by inserting segments with known "true-patterns" using the script `preprocess_data_records.m`. The original data records were part of the infant data described in [1].

14) RIPScore will save a backup of the analysis every 5 min.

IV. Configuration

By default, RIPScore is configured to start in Blind Scorer Mode, a mode that randomly selects the next record to analyze, from a directory containing sample data records. For project specific needs it may be necessary to use alternative configurations. There are 2 ways to trigger RIPScore's configuration process, which are:

1) Run the function `RIPScore_setParameters` with parameter `'C:\McCRIB\McCRIBS\RIPSCORE\RIPScore'`, or
2) Delete the file `C:\McCRIB\McCRIBS\RIPSCORE\RIPScore\cnf.mat` and run `RIPScore`.

This will initialize an interactive configuration process where RIPScore will ask the questions described next.

1) Select RIPScore Mode:

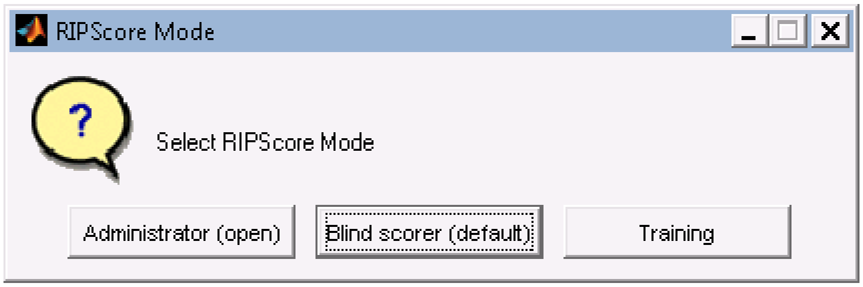

Set the Mode in which the application will run.

- `Administrator (open)`: RIPScore runs in Scorer mode; it lets the user load any RIPScore data file. This mode can be used for non-randomized scoring, and to review the scores from different scorers.
- `Blind scorer (default)`: RIPScore runs in Scorer mode; it keeps the file selection blinded from the user, by automatically, and randomly selecting the next file to analyze.
- `Training`: RIPScore runs in Training mode; it generates simulated data, provides practice with interactive feedback for the user, and evaluates the user performance.

2) Scoring results path:

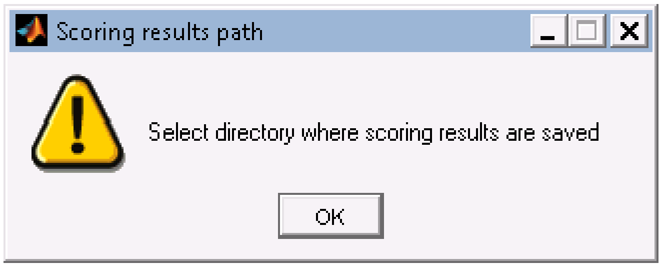

Choose/create a directory where the scoring analysis results will be saved. In the default configuration, this directory is `C:\McCRIB\McCRIBS\RIPSCORE\scored`. After selecting the directory, RIPScore will output the full path to it:

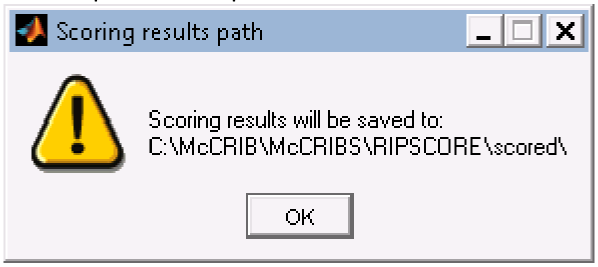

3) Data records path:

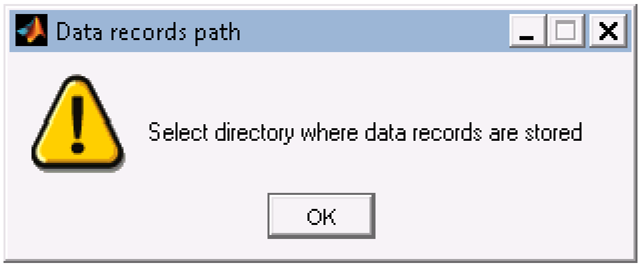

Choose/create a directory where the raw data to be analyzed is saved. In the default configuration, this directory is `C:\McCRIB\McCRIBS\RIPSCORE\data`. After selecting the directory, RIPScore will output the full path to it:

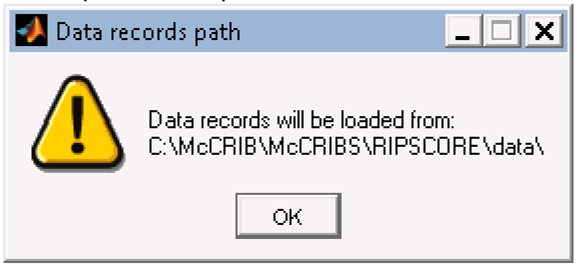

4) Set Environment Variables:

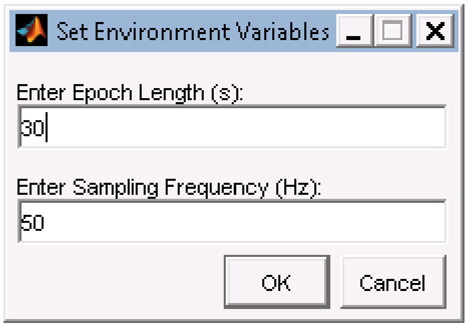

Enter the length of the data epoch displayed on the screen (default is 30 s), and the sampling frequency of the data (default is 50 Hz). Click `OK`.

5) Set Training Mode Variables:

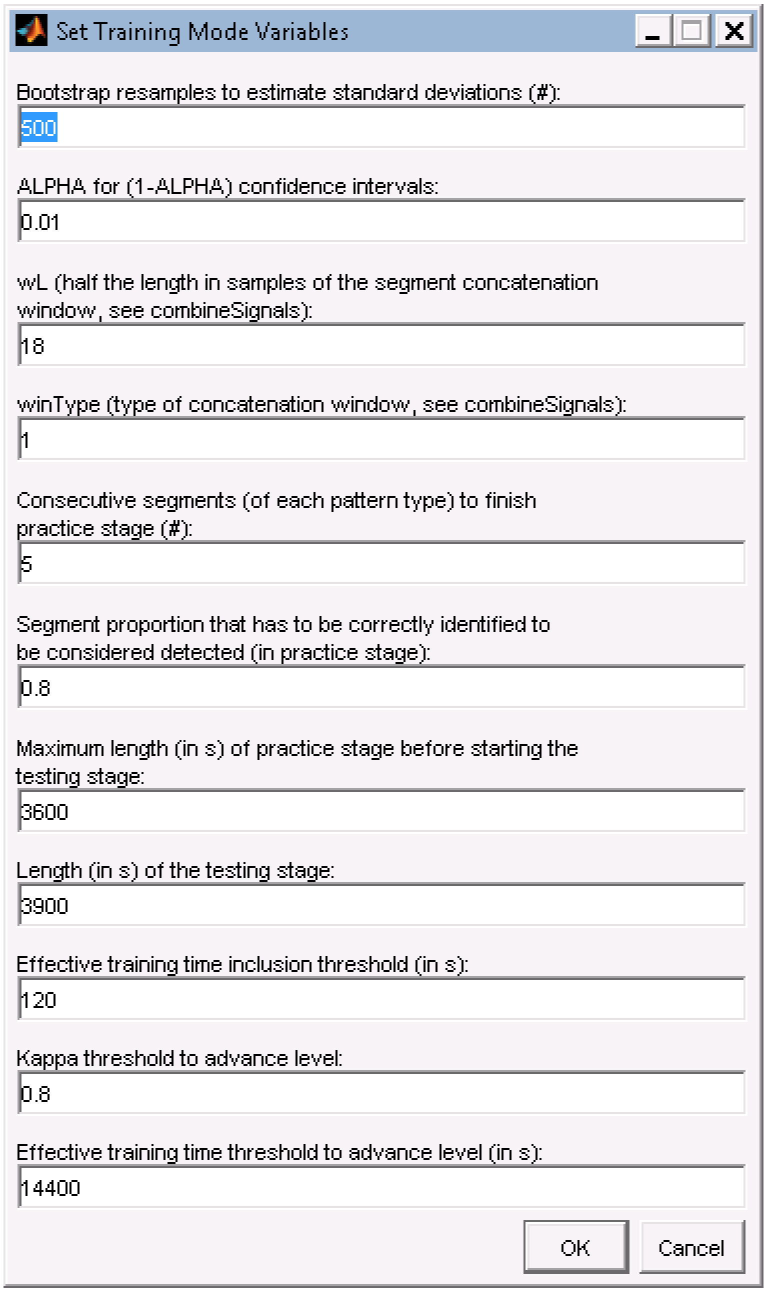

This is only required if RIPScore is set to run in Training Mode (just click `OK` for any other mode). RIPScore will suggest the default values. The variables are:

- `Bootstrap resamples (#)`: This is an integer used to estimate standard deviations of kappa estimates.
- `ALPHA`: This is a real value in the open interval (0, 1), used for computation of (1-`ALPHA`) confidence intervals.
- `wL (samples)`: This is an integer with half the length of the segment concatenation window; see function `combineSignals` for additional information.
- `winType`: This is an integer with the type of the concatenation window; see function `combineSignals` for additional information.

- Consecutive segments (#): This is an integer with the number of consecutive segments, of each pattern type, that are required to finish practice stage.
- Segment proportion: A real value in the interval (0,1] that indicates the proportion of a scored segment that has to match the Actual Pattern to be considered correct (in practice stage).
- Maximum length of practice stage (s): The maximum length of the practice stage before the start of the testing stage.
- Length of the testing stage (s): The length of the testing stage.
- Effective training time inclusion threshold (s): The maximum time difference between consecutive scored segments' timestamps to be included in the calculation of effective training time.
- Kappa threshold: The minimum kappa required to advance from level 1 to level 2.
- Effective training time threshold to advance level (s): The maximum effective training time permitted when advancing from level 1 to level 2.

6) Select the "true-pattern" segment library file:

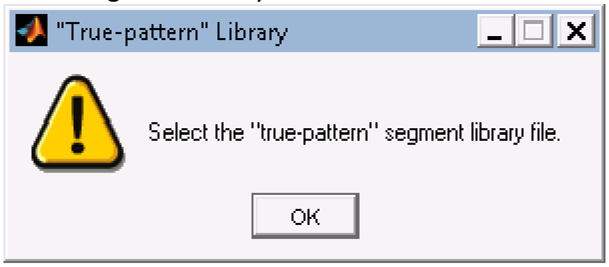

This is only required if RIPScore is set to run in Training Mode (click `OK` and then `Esc` for any other mode). Choose the file containing the library of "true-pattern" segments. In the default configuration, this file is:
`C:\McCRIB\Data\POA\TruePattern_Segment_Library\TruePattern_Library.mat`.
After selecting the file, RIPScore will output the full path to it:

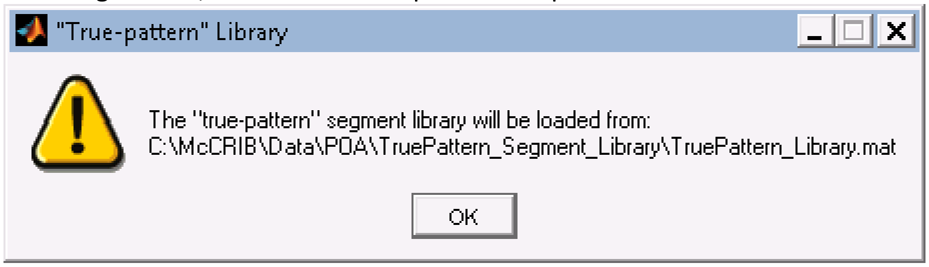

7) Click `OK`. RIPScore is now configured, and will save a new `cnf.mat` file in the application's main directory.

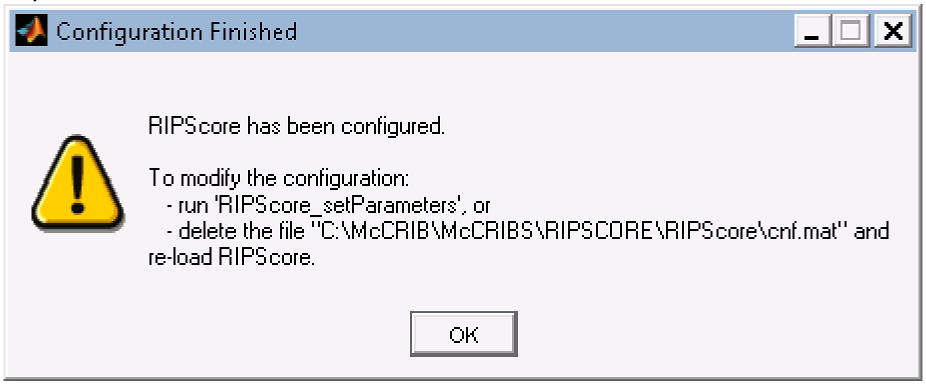

V. Data Files

This section describes the format of files used by RIPScore; it is divided in input and output files.

A. Input

RIPScore reads cardiorespiratory data stored in MATLAB™ *.mat format. An input file must have the following variable:

- `data`: An N-by-5 matrix with the recorded raw data. The columns have the following signals: (1) time (s), (2) ribcage (a.u.), (3) abdomen (a.u.), (4) photoplethysmography (a.u.), and (5) blood oxygen saturation (%).

B. Output

RIPScore generates several files related to the training of scorers and their analyses. The files are in MATLAB™ *.mat format. The following list describes the contents of these files.

1) Scorer data (e.g., `scorer_TEST.mat`).
   This file has a summary of the files analyzed by scorer `TEST`. The file contains the variables described next.
   - `SCORER`: A struct with the following fields:
     - `finished`, a struct with a list of all files that have been completely scored. It has the following fields:
       - `file`, a 1-by-F cell array of strings, where each cell has the name of a file that has been fully scored.
       - `alias`, a 1-by-F cell array of strings that lists the aliases for the files listed in `SCORER.finished.file`.
     - `current`, a struct with the following fields:
       - `file`, a string with the name of the file that is currently being scored.
       - `alias`, the alias assigned to the file that is currently being scored.
     - `RIPScoreVersion`, a string with the version of RIPScore that created this file.

2) Scoring results (e.g., `scored_TEST_ALIAS.mat`).
   This file has the scoring details from an analysis performed by scorer `TEST`. The term `ALIAS` in the file name corresponds to the alias of the scored file with name listed in `SCORER.finished.file` or `SCORER.current.file`. This file contains the variables described next.
   - `SCORING`: A struct with the detailed results from the manual analysis. It has the following fields:
     - `NextScore`, an index indicating the number of segments scored (S) plus 1.
     - `Events`, an S-by-4 matrix with the RIP patterns assigned to each data segment. The columns are: (1) segment start time, (2) segment end time, (3) code of pattern assigned to the segment (pause=1, asynchronous-breathing=2, movement artifact=3, synchronous-breathing=4, sigh=5, unknown=99), (4) timestamp.
     - `Scorer`, a string with the scorer ID.
     - `Comments`, a struct with comments that the scorer assigned to scored segments. It has the following fields:
       - `start`, a C-by-1 vector with the start time of the segment linked to the comment.
       - `end`: a C-by-1 vector with the end time of the segment linked to the comment.

        - `eventID`: a C-by-1 vector with the index of the linked segment as it appears in `SCORING.Events`.
        - `comment`: a 1-by-C cell array with the comments assigned to the linked segments.
    - `ElapsedTime`, deprecated.
    - `Completed`, a flag indicating whether this data record has been completely scored or not.
    - `FileName`, a string with the name of this file.
    - `RIPScoreVersion`, a string with the version of RIPScore that created this file.
    - `currentTime`, a scalar with the latest Epoch Start Time.

3) Trainee data (e.g., `trainee_TEST.mat`).
   This is a file with a summary of the training results from scorer `TEST`. The file contains the variables described next.
   - `TRAINEE`: A struct with the following fields:
     - `Scorer`, a string with the scorer's ID.
     - `level`, an integer indicating the latest scorer's training level.
     - `iteration`, a 1-by-2 vector with the last session completed in level 1 (col1) and level 2 (col2).
     - `InterAgreement`, a 1-by-2 cell array with estimates of the accuracy performance (estimated using the Fleiss' kappa statistic [2, 3]) in level 1 (col1) and level 2 (col2). Each cell is a struct array with the following fields:
       - `k`, a 1-by-M vector with the kappa estimate from each of the M training sessions.
       - `kj`, an M-by-6 vector with the pattern-specific kappa estimate from each of the M training sessions. Each column corresponds to one the 6 unique pattern types: col1=Pause, col2=Asynchronous-breathing, col3=Movement artifact, col4=Synchronous-breathing, col5=Sigh, col6=Unknown.
       - `kstd`, a 1-by-M vector with the estimated standard deviation of kappa for the M training sessions. RIPScore estimates standard deviations using the bootstrap method [4].
       - `kjstd`, an M-by-6 vector with the estimates of pattern-specific standard deviation of kappa for each of the M training sessions. RIPScore estimates standard deviations using the bootstrap method. Each column corresponds to one the 6 unique pattern types: col1=Pause, col2=Asynchronous-breathing, col3=Movement artifact, col4=Synchronous-breathing, col5=Sigh, col6=Unknown.
       - `kcil`, deprecated.
       - `kcih`, deprecated.
       - `kjcil`, deprecated.
       - `kjcih`, deprecated.
     - `IntraAgreement`, a 1-by-2 cell array with estimates of the consistency performance (estimated using the Fleiss' kappa statistic [2, 3]) in level 1 (col1) and level 2 (col2). Each cell is a struct array with the following fields:
       - `k`, a 1-by-M vector with the kappa estimate from each of the M training sessions.
       - `kj`, an M-by-6 vector with the pattern-specific kappa estimate from each of the M training sessions. Each column corresponds to one the 6 unique pattern types: col1=Pause, col2=Asynchronous-breathing, col3=Movement artifact, col4=Synchronous-breathing, col5=Sigh, col6=Unknown.

        - kstd, a 1-by-M vector with the estimated standard deviation of kappa for the M training sessions. RIPScore estimates standard deviations using the bootstrap method [4].
        - kjstd, an M-by-6 vector with the estimates of pattern-specific standard deviation of kappa for each of the M training sessions. RIPScore estimates standard deviations using the bootstrap method. Each column corresponds to one the 6 unique pattern types: col1=Pause, col2=Asynchronous-breathing, col3=Movement artifact, col4=Synchronous-breathing, col5=Sigh, col6=Unknown.
        - kcil, deprecated.
        - kcih, deprecated.
        - kjcil, deprecated.
        - kjcih, deprecated.
    - ScoredSamples, a 1-by-2 cell array with the number of samples scored in each session. Col1=Level 1, Col2=Level2. Each cell is a 1-by-M vector where M is the number of training sessions.
    - PracticeTime, a 1-by-2 cell array with the time (in seconds) required to complete practice in each session. Col1=Level 1, Col2=Level2. Each cell is a 1-by-M vector where M is the number of training sessions.
    - IterationTime, a 1-by-2 cell array with the time (in seconds) required to complete each session. Col1=Level 1, Col2=Level2. Each cell is a 1-by-M vector where M is the number of training sessions.
    - savepath, a string with the path to the directory where this file is saved.
    - savename, a string with the name of this file.
    - RIPScoreVersion, a string with the version of RIPScore that created this file.
    - WorkingSession, a struct with the following runtime fields:
        - isActive, a flag indicating if the trainee hasn't finished the current session (0=finished, 1=not finished).
        - fileSaved, a string with the path and name of the latest SAVED version of the current training session.
        - fileBackup, a string with the path and name of the latest BACKUP version of the current training session.
        - fileSelected, a string with the path and name of the file selected to be loaded if there are both SAVED and BACKUP versions. If there are neither SAVED nor BACKUP versions this variable is an empty string.
    - ConfusionMatrix, a 2-by-M cell array with estimates of the scorer's confusion matrix from each training session. The first row corresponds to level 1 and the second to level 2. Each column corresponds to each session. Each cell contains a 6-by-6 matrix where the rows correspond to the patterns assigned by the scorer and the columns to the actual pattern. Col1/row1=Pause, col2/row2=Asynchronous-breathing, col3/row3=Movement artifact, col4/row4=Synchronous-breathing, col5/row5=Sigh, col6/row6=Unknown.
    - EffectiveTrainingTime, a 1-by-2 cell array with the effective time (in seconds) required to complete the training session (see [1]). Col1=Level 1, Col2=Level2. Each cell is a 1-by-M vector where M is the number of training sessions.

4) Training session results (e.g., trained_TEST_level_X_iteration_Y.mat).

This is a file with the scoring details from a training session of scorer `TEST`. The name indicates that it corresponds to the `Y`th training session on level `X`. The file contains the variables described next.

- `SCORING`: A struct with the detailed results from the manual analysis. It has the following fields:
  - `NextScore`, an index indicating the number of segments scored (S) plus 1.
  - `Events`, an S-by-4 matrix with the patterns assigned to each data segment. The columns are: (1) segment start time, (2) segment end time, (3) code of pattern assigned to the segment (pause=1, asynchronous-breathing=2, movement artifact=3, synchronous-breathing=4, sigh=5, unknown=99), (4) timestamp.
  - `Scorer`, a string with the scorer ID.
  - `Comments`, a struct with comments that the scorer assigned to scored segments. It has the following fields:
    - `start`, a C-by-1 vector with the start time of the segment linked to the comment.
    - `end`: a C-by-1 vector with the end time of the segment linked to the comment.
    - `eventID`: a C-by-1 vector with the index of the linked segment as it appears in `SCORING.Events`.
    - `comment`: a 1-by-C cell array with the comments assigned to the linked segments.
  - `ElapsedTime`, deprecated.
  - `Completed`, a flag indicating whether this data record has been completely scored or not.
  - `FileName`, a string with the name of this file.
  - `RIPScoreVersion`, a string with the version of RIPScore that created this file.
  - `currentTime`, a scalar with the latest Epoch Start Time.

- `DATA`: A struct with the data used in this training session. It has the following fields:
  - `RC`, an N-by-1 vector with the ribcage signal generated for this session.
  - `AB`, an N-by-1 vector with the abdomen signal generated for this session.
  - `PP`, an N-by-1 vector with the photoplethysmography signal generated for this session.
  - `SA`, an N-by-1 vector with the blood oxygen saturation signal generated for this session.
  - `State`, an N-by-1 vector with the actual pattern for each sample in `DATA`.
  - `EventID`, an N-by-1 vector with the ID of the segment each sample in `DATA` belongs to.
  - `IsTestSegment`, an N-by-1 vector zero-valued for samples in the training stage, and one-valued for samples in the evaluation stage.
  - `RIPScoreVersion`, a string with the version of RIPScore that created this file.

VI. List of Acronyms

McCRIBS	McGill CardioRespiratory Infant Behavior Software
RIP	Respiratory Inductive Plethysmography

VII. References

[1] Robles-Rubio CA, Bertolizio G, Brown KA and Kearney RE (2015) Scoring Tools for the Analysis of Infant Respiratory Inductive Plethysmography Signals. Submitted to PLoS One.
[2] Fleiss JL (1971) Measuring nominal scale agreement among many raters. Psychol Bulletin 76: 378-382.
[3] Cardillo G (2007) Fleiss'es kappa: compute the Fleiss'es kappa for multiple raters. MATLAB CENTRAL: The MathWorks, Inc.
[4] Efron B and Tibshirani RJ (1993) An introduction to the bootstrap. New York [etc.]: Chapman & Hall.
